# Non-Contact Dual-Laser Doppler Vibrometry for Synchronous Sensing of Vibration Response Evolution and Collapse Precursors of Unstable Rock Mass Under Repeated Aftershock Impacts

**DOI:** 10.3390/s26217091

**Published:** 2026-11-09

**Authors:** Ruijie Xi, Shaobo Chai, Yongqiang Zhou, Juehao Huang, Xiaodong Fu, Tingjin Deng

**Affiliations:** 1 School of Civil Engineering and Smart Construction, Chang’an University, Xi’an 710064, China; 2024128041@chd.edu.cn (R.X.);; 2 State Key Laboratory of Geomechanics and Geotechnical Engineering Safety, Institute of Rock and Soil Mechanics, Chinese Academy of Sciences, Wuhan 430071, China; jhhuang@whrsm.ac.cn (J.H.);; 3 School of Engineering Science, University of Chinese Academy of Sciences, Beijing 100049, China

**Keywords:** laser Doppler vibrometer (LDV), non-contact optical vibration sensing, dual-channel synchronous monitoring, unstable rock mass, aftershock, structural health monitoring, collapse precursor

## Abstract

**Highlights:**

**What are the main findings?**

- A non-contact dual-LDV synchronous sensing scheme using a bedrock reference channel separates external impact excitation from the rock’s own vibration and jointly resolves time-domain, frequency-domain, and cross-channel coupling indicators.
- Repeated impacts drive an end-of-life threshold jump in unstable-rock vibration, while the dominant frequency remains insensitive to interface degradation.

**What are the implications of the main findings?**

- Synchronous dual-LDV monitoring provides a non-contact approach to tracking vibration evolution in unstable rock.
- Combining amplitude anomalies with cross-channel coupling supports a graded warning framework for identifying potential precursory changes.

**Abstract:**

After a mainshock, unstable rock masses often collapse with a time lag under repeated aftershocks, while conventional contact sensors struggle to capture high-frequency micro-amplitude pre-collapse responses on inaccessible high-steep slopes, and existing single-point laser Doppler vibrometry (LDV) cannot distinguish external excitation from structural degradation. This study proposes a non-contact dual-LDV synchronous sensing scheme with a bedrock reference channel and an unstable-rock measurement channel. Ten collapse tests were conducted on a cement-block model with an ice-bonded interface using repeated ball-drop impacts to simulate aftershocks, and time-domain, frequency-domain, and cross-channel coupling indicators were extracted from 114 effective segments. The results show that all specimens failed after 6–21 impacts; the zero-lag correlation deficit increases first in the progressive stage, and normalized *RMS* jumps sharply before collapse; the dominant frequency remains stable at 10–11 Hz and is insensitive to interface damage; and only six specimens show persistent 3σ *RMS* precursors, while the multi-indicator fusion framework effectively suppresses false and missed alarms from single thresholds. The proposed scheme provides an experimental basis and a multi-indicator analytical framework for non-contact early warning of aftershock-driven unstable rock collapse.

## 1. Introduction

**Figure 1:**
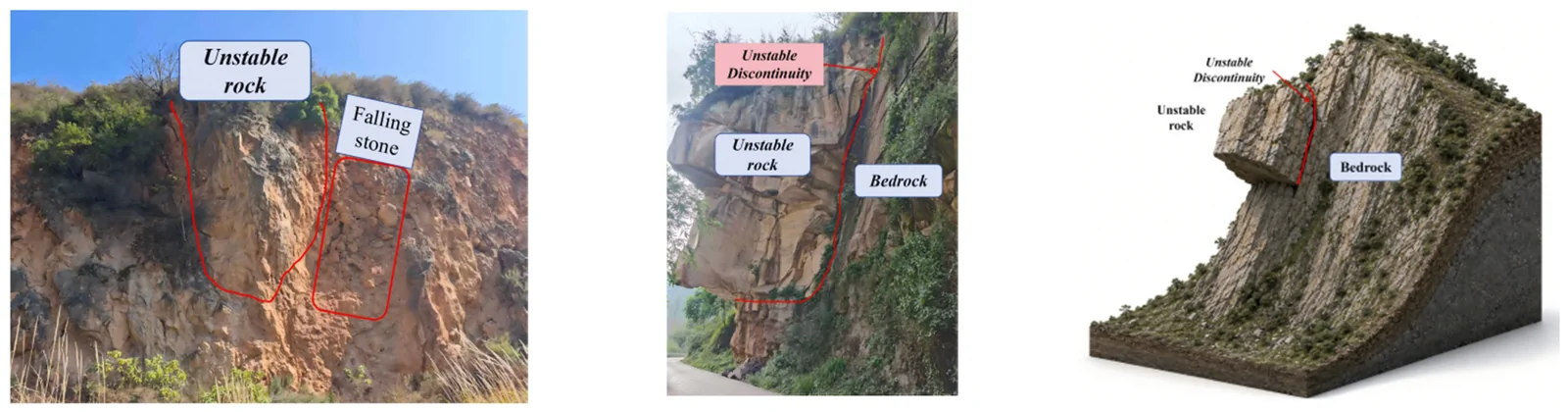
Physical illustration of the bedrock and the unstable rock mass.

High-steep rock slopes are widely distributed in the seismically active mountainous areas of China, where earthquakes, aftershocks, and traffic vibration constitute frequent dynamic disturbances [1]. The unstable rock masses on these slopes commonly contain pre-existing fractures and damaged structural planes, so their stability is highly sensitive to external dynamic loading; sustained or repeated disturbance drives fracture propagation and structural-plane degradation, progressively raising the probability of failure and collapse that threatens highways, railways, and residents’ lives and property [2] (Figure 1). Reliably sensing the internal deterioration of such unstable rock masses before collapse—especially at high positions that personnel cannot reach—has therefore become a central problem in slope structural health monitoring, in which non-contact, high-rate vibration measurement is expected to play a key role.

Dynamic loading is the principal external driver of structural degradation and collapse of unstable rock, encompassing both instantaneous strong-impact failure and long-term dynamic fatigue. As a typical strong dynamic load, earthquakes promote tensile opening and shear dislocation of structural planes through the propagation, reflection, and superposition of seismic waves, continuously weakening the overall stability of the rock mass. Using particle-flow numerical simulation, Tian et al. established a dynamic model of layered unstable rock and showed that, as seismic acceleration increased, the stability coefficient fluctuated sharply and decreased continuously, with the horizontal component far more damaging than the vertical component [3]. Tang et al. combined shaking-table tests and numerical simulation for slopes with different structural planes and found that repeated reflection of seismic waves between structural planes and the slope surface in layered slopes produces a marked amplification effect [4]. Huang et al. further showed that the failure of toppling unstable rock under earthquakes is essentially tensile failure: cracks initiate at the peak tensile displacement and rapidly coalesce, while multi-directional wave superposition intensifies the strain energy released during collapse [5]. For anti-dip slopes containing weak interlayers, Zhao et al. reported a bottom-up progressive damage pattern under cyclic loading [6].

To reveal the vibration response and progressive failure of unstable rock under dynamic loading, researchers have conducted extensive work through three complementary routes—field monitoring, numerical simulation, and physical model tests—establishing the intrinsic relationship between vibration characteristics and structural state and thereby providing the methodological basis for sensor-based collapse early warning.

Among these routes, field monitoring first adopted ambient micro-vibration and resonance-frequency measurements to sense unstable rock slopes. Burjánek et al. performed ambient vibration tests on the unstable Randa rock slope in Switzerland, identified characteristic modes related to stability, and demonstrated for the first time that ambient vibration can effectively reflect the dynamic properties of unstable rock [7]. Bottelin et al. applied ambient vibration spectral analysis to prone-to-fall rock compartments and found that unstable blocks possess stable characteristic resonance frequencies that vary regularly with structural damage and can therefore characterize the evolution of structural state [8]. Burjánek et al. then carried out long-term ambient vibration monitoring on a slope approaching failure and showed that vibration characteristics sensitively reflect changes in structural state and boundary conditions, offering direct field evidence of the pre-collapse dynamic response [9]. On the Gondo slope, Kleinbrod et al. measured vibration amplification factors as high as 70 in the unstable zone, with these amplification characteristics remaining stable as the structure changed, indicating good robustness of ambient-vibration sensing [10].

Long-term continuous monitoring and multi-parameter joint analysis have since become a major field direction. Häusler et al. applied resonance mode analysis to long-term monitoring of the Preonzo unstable rock mass, obtaining complete modal parameters such as fundamental frequency, damping ratio, and mode shape, and showed that ambient vibration monitoring complements displacement monitoring by revealing internal damage evolution [11]. From more than a decade of passive seismic monitoring at the La Praz unstable rock mass in the French Alps, Bottelin and Baillet found that its fundamental frequency dropped by about 24% in the six months before collapse and that combining frequency evolution with deformation could distinguish brittle fracture from progressive sliding, confirming the value of long-term dynamic sensing for detecting stiffness degradation [12]. Field monitoring has also shifted from displacement toward dynamic response: Xu et al. used microseismic monitoring to show that internal micro-fracture activity and its spatiotemporal evolution respond to rock damage and construction disturbance, supplementing conventional deformation monitoring [13]. Liu et al. examined the dynamic cumulative damage of unstable rock slopes under water-level-fluctuation-zone degradation coupled with seismic action, revealing gradual damage accumulation toward failure [14]. Chen et al. further constructed a dynamic monitoring and early-warning system for unstable rock collapse and demonstrated its engineering application [15]. Overall, field monitoring has fully confirmed that vibration parameters are sensitive to structural damage, yet the complex field environment—wind loads, traffic vibration, and other noise—easily overlaps with structural signals, demanding high anti-interference capability from the sensing and signal-processing scheme.

Numerical simulation, with controllable and repeatable loading and access to internal stress states, has become an important complement to field measurement. Through distinct-element modeling, Jensen and Moore studied the evolution of resonance frequencies under different failure modes and found that the development of open fractures is generally accompanied by a decrease in fundamental frequency, but that different modes yield differentiated frequency responses, indicating that resonance frequency is not a universal criterion and that multi-indicator analysis is required [16].

Physical model tests can reproduce the complete damage-to-failure process under fully controlled conditions and are therefore key to revealing collapse precursors and validating field and numerical results. From slope microtremor signals, Yang et al. found that pre-collapse microtremor energy rises markedly and the dominant frequency shifts toward lower values, providing time- and frequency-domain precursor evidence [17]. In laboratory tests, Du et al. likewise observed increasing vibration amplitude and decreasing dominant frequency before failure; they developed safety monitoring tests based on natural vibration frequency, showed that reduced bonding of structural planes alters the dynamic properties and that dominant frequency can quantify stability, and used freeze–thaw physical simulation to capture abrupt changes in both amplitude and dominant frequency in the separation and accelerated-failure stages [18,19,20]. Jia et al. established stability evaluation models for sliding- and falling-type dangerous rock blocks, revealing differentiated frequency responses under different failure modes [21].

Despite this progress, field monitoring, numerical simulation, and conventional physical model tests still leave clear gaps for high-precision pre-collapse sensing. Field measurements struggle to separate external dynamic excitation from the rock’s own structural vibration, so environmental noise is easily misread as damage; numerical results depend strongly on the constitutive model and mechanical parameters and inevitably deviate from real damage evolution; and traditional physical model tests rely mostly on contact sensors, whose sampling rate and installation limit their ability to capture high-frequency, micro-amplitude vibrations near critical failure.

As a non-contact optical sensor, the laser Doppler vibrometer (LDV) offers high precision and high spatiotemporal resolution without requiring access or added mass, and has increasingly been applied to vibration sensing of rock slopes and structures. Saito et al. developed a remotely positioned LDV system suitable for field use and achieved, for the first time, non-contact vibration measurement of rock slopes [22]. Du et al. used LDV to experimentally study the failure process of rock blocks and proposed an early-warning indicator based on dominant frequency [23]. Ma et al. verified through model tests the correlation between the LDV-measured blocked vibration frequency and rock stability, revealing an inverse relationship between block size and vibration frequency [24]. Fukata et al. proposed a rockfall risk estimation method based on vibration measurement, advancing LDV sensing from qualitative identification toward quantitative assessment [25]. In the past two years, the application of LDV in slope dynamic monitoring has been further extended toward engineering practicability. Some studies have integrated LDV with mobile carrying platforms to realize rapid non-contact vibration scanning of inaccessible high-steep rock slopes, while other works have developed adaptive signal denoising algorithms targeting field interference factors such as wind disturbance and atmospheric turbulence, which effectively improve the environmental adaptability of LDV monitoring technology. Despite these advances, existing LDV applications for rock slopes have predominantly relied on single-point measurements.

Single-point LDV measures only one surface and cannot distinguish whether an observed vibration increase originates from a stronger external excitation or from genuine structural degradation. In contrast, a dual-channel synchronous configuration records the bedrock and the unstable rock simultaneously: the bedrock acts as a stable reference channel that characterizes the common excitation input, while the unstable-rock channel serves as the measurement channel. Their relative motion, cross-channel coupling, and dynamic transmission can then be extracted to sense interface degradation, but the potential of this reference-channel design for collapse early warning remains largely unexplored.

Accordingly, three sensing-level problems remain unsolved. First, it is difficult to effectively separate external dynamic excitation from the structural vibration of the unstable rock itself; most studies analyze the two in a mixed manner, which obscures the source of the dynamic response and lets precursor identification be interfered with by external forces. Second, existing studies mostly rely on single indicators such as amplitude, dominant frequency, or energy, which cannot fully portray the multidimensional pre-collapse evolution and are vulnerable to environmental noise and random disturbance, limiting warning reliability. In fact, the multi-parameter fusion early warning strategy has been widely verified in geotechnical dynamic disaster monitoring. For scenarios such as deep coal seam rockburst and underground thermal storage cavity stability assessment, relevant studies have confirmed that fusing multi-dimensional temporal dynamic features can effectively reduce the false alarm and missed alarm risks of single indicators, and significantly improve the robustness of disaster early warning [26,27]. However, such a fusion framework combining dual-channel coupling features with single-channel amplitude and frequency indicators has not yet been established for LDV-based unstable rock collapse monitoring. Third, the relative vibration response between the bedrock reference and the unstable-rock measurement point has not been fully exploited for sensing interface deterioration, especially the cross-channel coupling that carries information about transmission and interface conditions.

**Figure 2:**
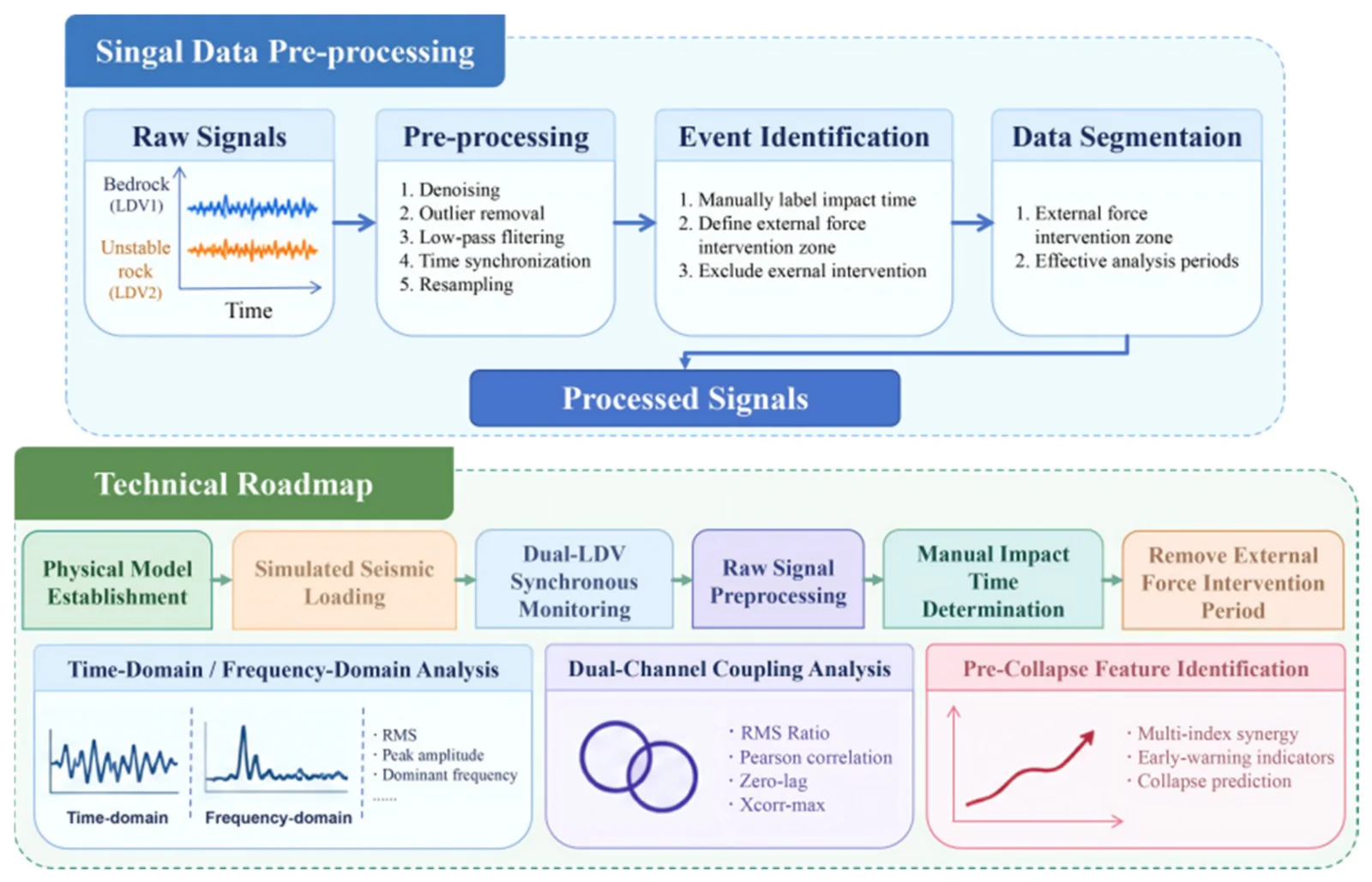
Research workflow and multi-indicator analysis framework of the study.

To address these problems, this study conducts small-scale physical model tests in which ball-drop impacts simulate external dynamic loading and a dual-LDV synchronously records the bedrock and unstable-rock channels; the technical route is shown in Figure 2. After constructing the physical model and debugging the loading system, the raw signals were denoised and time-synchronized, the ball-loading moments were manually marked, and the direct external-disturbance intervals were excluded so as to distinguish the forced response during impacts from the free response in the inter-impact intervals. Time-domain and frequency-domain features (normalized *RMS*, peak frequency, and peak amplitude) and cross-channel coupling features (*RMS* ratio, sliding Pearson correlation, zero-lag correlation, and maximum cross-correlation) were then extracted, and their anomalous evolution before collapse was identified through temporal trend and statistical analysis.

On this basis, the main contributions of this work, viewed from the sensor and signal-processing perspective, are summarized as follows:

- A non-contact dual-LDV synchronous sensing architecture is established, pairing a bedrock reference channel with an unstable-rock measurement channel so that the common excitation input and the local structural response can be acquired simultaneously, providing the data basis for interface dynamic-coupling analysis.
- An explicit disturbance-period segmentation method is introduced: By excluding the direct-impact intervals before and after each ball loading, external excitation is separated from the rock’s autonomous vibration, offering a practical scheme for distinguishing the source of the dynamic response.
- A multi-indicator co-evolution analysis framework is proposed that fuses time-domain, frequency-domain, and cross-channel coupling indicators, reducing the uncertainty of single-indicator interpretation and improving the completeness of pre-collapse anomaly identification.

The results provide experimental evidence and a methodological basis for improving the early-warning indicator system of unstable rock collapse, and demonstrate a deployable non-contact sensing scheme for high-steep slopes in alpine, earthquake-prone regions. The remainder of this paper is organized as follows: Section 2 describes the physical model, the dual-LDV sensing configuration, and the repeated-impact loading scheme; Section 3 presents the time-domain, frequency-domain, and cross-channel coupling results; Section 4 discusses the controlling effect of aftershock number, the difference from natural-melting controls, and the multi-indicator co-evolution and its engineering early-warning significance; and Section 5 concludes the paper.

## 2. Test Materials and Methods

### 2.1. Establishment of the Physical Model

**Figure 3:**
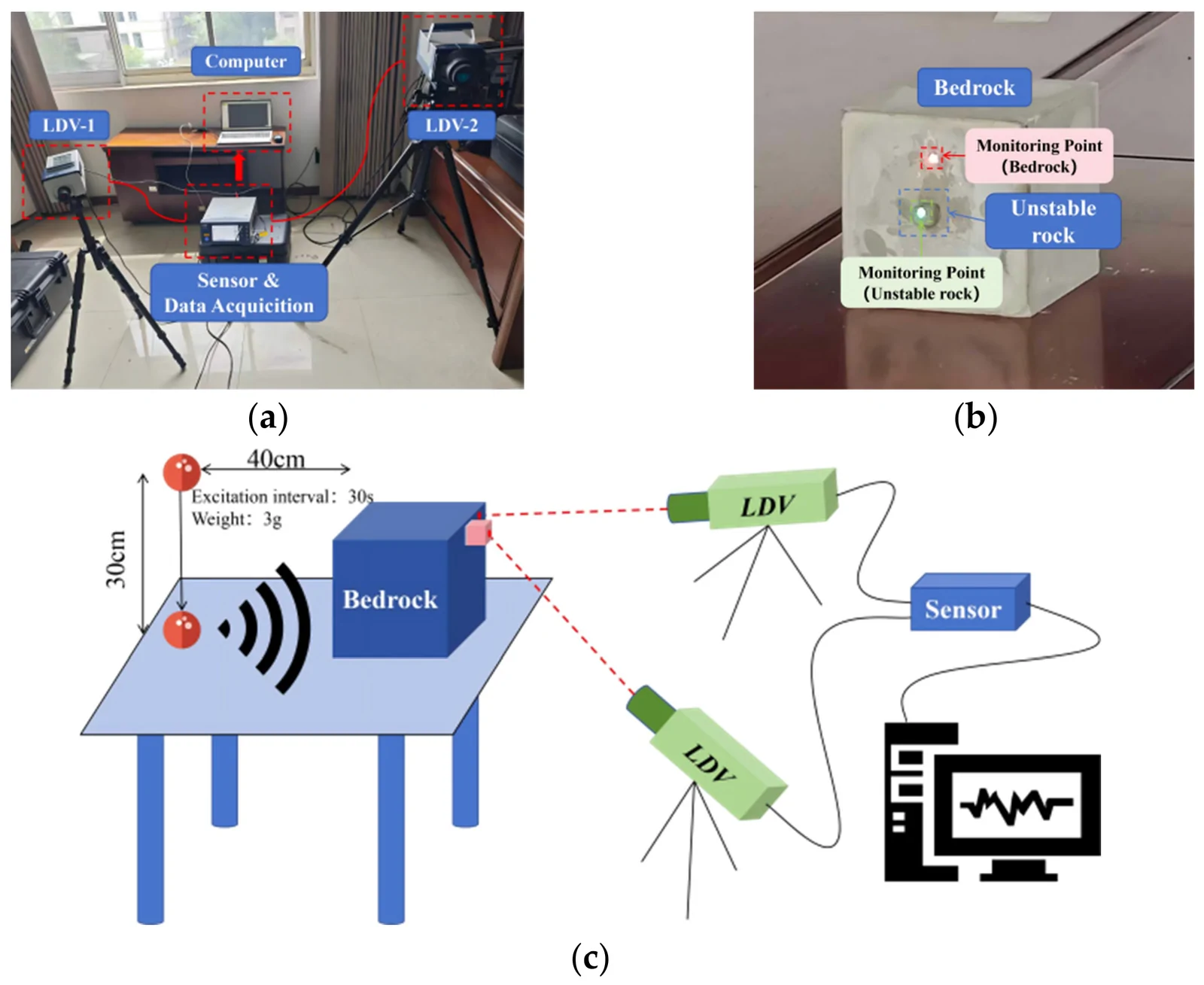
Experimental setup, bedrock–unstable rock physical model, and dual-LDV monitoring configuration: (**a**) experimental equipment; (**b**) bedrock–unstable rock physical model; (**c**) schematic of synchronous dual-LDV monitoring and repeated aftershock impact loading.

In natural high-steep rock slopes, unstable rock masses mostly attach to the surface of intact bedrock in the form of local blocks, and their failure process is controlled by the progressive degradation of the dominant structural plane. The whole system presents the dynamic characteristics of a large stable base, a locally dangerous block, and a weak connecting interface. To accurately reveal the evolution of the vibration response of unstable rock during interface degradation, this test followed the physical simulation principle of retaining the dominant mechanisms and simplifying secondary factors, and generalized the field rock connection into an indoor model from the two aspects of scale and interface(Figure 3).

(1) Geometric and mass scale. The physical model was designed to reproduce the essential configuration of a stable bedrock, a small unstable rock block, and a progressively deteriorating interface (Figure 3). Homogeneous precast cement-mortar blocks were used to represent the bedrock and unstable rock mass, with dimensions of 10 cm × 10 cm × 10 cm and 1 cm × 1 cm × 1 cm, respectively. The characteristic length ratio of the unstable block to the bedrock was therefore 1:10, while the volume ratio was 1:1000. Assuming the same material density, the corresponding mass ratio was also approximately 1:1000. For geometrically similar blocks made of the same material, the characteristic bulk stiffness scales as *K*∝ *EL*; thus, the estimated stiffness ratio of the block bodies is approximately 1:10. The actual dynamic response of the assembled model is additionally governed by the interface stiffness, contact conditions, and energy dissipation. The measured mean peak frequencies were 10.93 Hz for the unstable block and 11.98 Hz for the bedrock (Table 1), providing the dynamic reference for the experimental configuration.

(2) Interface degradation mechanism. The degradation of natural unstable rock masses is commonly associated with progressive weakening of the dominant structural plane, including reductions in effective contact area and bonding strength. In this experiment, an ice interface was introduced between the unstable block and the bedrock to reproduce this progressive weakening process. The nominal contact footprint corresponded to the 1 × 1 cm^2^ basal face of the unstable block, giving an initial geometric contact area of 1 cm^2^. Under natural melting at room temperature, the effective bonded area and interface support progressively decreased, ultimately allowing the unstable block to detach when the remaining support could no longer sustain its weight. The ice interface therefore provided a controllable means of reproducing time-dependent interface degradation, while the larger bedrock block served as the reference for simultaneous vibration monitoring. The resulting model preserves the principal mass contrast and interface-controlled failure mechanism relevant to the study, with the measured vibration response used to characterize the evolution of the assembled system.

### 2.2. Test Layout and Multiple Simulated Aftershock Impact Loading Scheme

In a real earthquake disaster chain, unstable rock collapse often does not occur at the instant of the main shock but lags behind it and develops gradually under the repeated disturbance of subsequent aftershocks [28]. Following a mainshock, already-damaged unstable rock masses may continue to deteriorate under subsequent aftershocks. Natural aftershocks impose time-varying inertial loads that may cause pre-existing discontinuities to open, close, and slip, resulting in progressive damage accumulation. To investigate the evolution of vibration responses under repeated disturbances, this study employed repeated steel-ball impacts as a controlled laboratory loading protocol. Impacts were applied at fixed 30 s intervals from the same release position to maintain consistent nominal loading conditions throughout each test. This approach enables the cumulative response evolution of the unstable block interface system to be examined under repeated transient excitation. Although a ball impact differs from the multi-component, time-varying inertial excitation of a natural aftershock, the repeated-impact protocol provides a reproducible means of investigating vibration-response changes associated with progressive interface degradation.

The loading system consisted of a 3 g steel ball released from a fixed position 40 cm horizontally behind and 30 cm vertically above the impact point (Figure 3c). Based on the reported vertical elevation difference of 0.30 m, the corresponding gravitational potential-energy change was calculated as

(1) $$E_{g}=mgh=8.83\times 10^{-3}\;J$$

If this potential-energy change were converted entirely into translational kinetic energy, the corresponding ideal impact speed would be

(2) $$v_{\mathrm{ideal}}=\sqrt{2gh}=2.43\;m/s$$

with an associated momentum of

(3) $$p_{\mathrm{ideal}}=mv=7.28\times 10^{-3}\;\mathrm{kg}\cdot m/s$$

These calculations provide a reference estimate of the available energy and momentum scale of the impact. The actual impact velocity and transferred energy depend on the ball’s trajectory, possible rotational motion, energy dissipation, and rebound. The impact impulse is governed by the momentum change,

(4) J=ΔP

while the mean contact force also depends on the contact duration. As the present system records surface vibration velocity rather than contact force, the impact force–time history is not directly measured.

During each test, the two LDV channels were recorded continuously, and a ball-drop impact was applied every 30 s until the unstable rock mass collapsed. After testing, the impact times were identified from the recorded time histories, and the signals were segmented with reference to the impact events and collapse time. For each impact occurring at time *t*_i_, the interval from (*t*_i_ − 0.5) s to (*t*_i_ + 2.5) s was defined as the direct external-disturbance interval and was excluded from subsequent feature calculations. The remaining continuous intervals before collapse were treated as effective analysis segments. When a disturbance interval overlapped the collapse time, only the pre-collapse portion of the record was retained, and no post-collapse segment was counted. This procedure separates the immediate response to external impact from the inter-impact vibration response used to characterize progressive changes in the unstable rock interface system. The signal-processing workflow is illustrated in Figure 2. The experimental instruments and relevant parameters are detailed in Table 2.

To evaluate measurement uncertainty and the influence of data-processing settings, sensitivity analyses were conducted for background noise levels, filtering parameters, time-window selections, and channel time-lag search ranges. The results indicate that the key indicators remain generally stable across reasonable variations in processing parameters, while the measured signals are distinguishable from background noise. Combined with the shared acquisition clock and high temporal resolution of the dual-channel system, these findings suggest that the observed time delays are unlikely to be artifacts of synchronization errors or noise. Overall, the principal findings demonstrate good robustness against measurement noise and data-processing choices.

## 3. Experimental Results and Analysis

Based on the dual-LDV synchronous vibration measurement data, this section systematically analyzes the evolution characteristics of the vibration response of each unstable rock mass in groups A1-A10 from the start of loading to before collapse and from three aspects: the time domain, the frequency domain, and dual-channel coupling.

### 3.1. Evolution of Time-Domain Characteristics

#### 3.1.1. Raw Time-Domain Signals and Loading Event Identification

To avoid the direct forced vibration produced by impacts interfering with the analysis of the structural response of the unstable rock itself, loading event identification was first performed on the raw time-domain signals of each test group, and the direct external-disturbance intervals were divided according to the manually marked ball-impact moments. Each test applied external dynamic disturbance in the form of periodic ball impacts and continued until the unstable rock finally collapsed. The collapse moments and impact times of groups A1–A10 are shown in Table 3.

**Figure 4:**
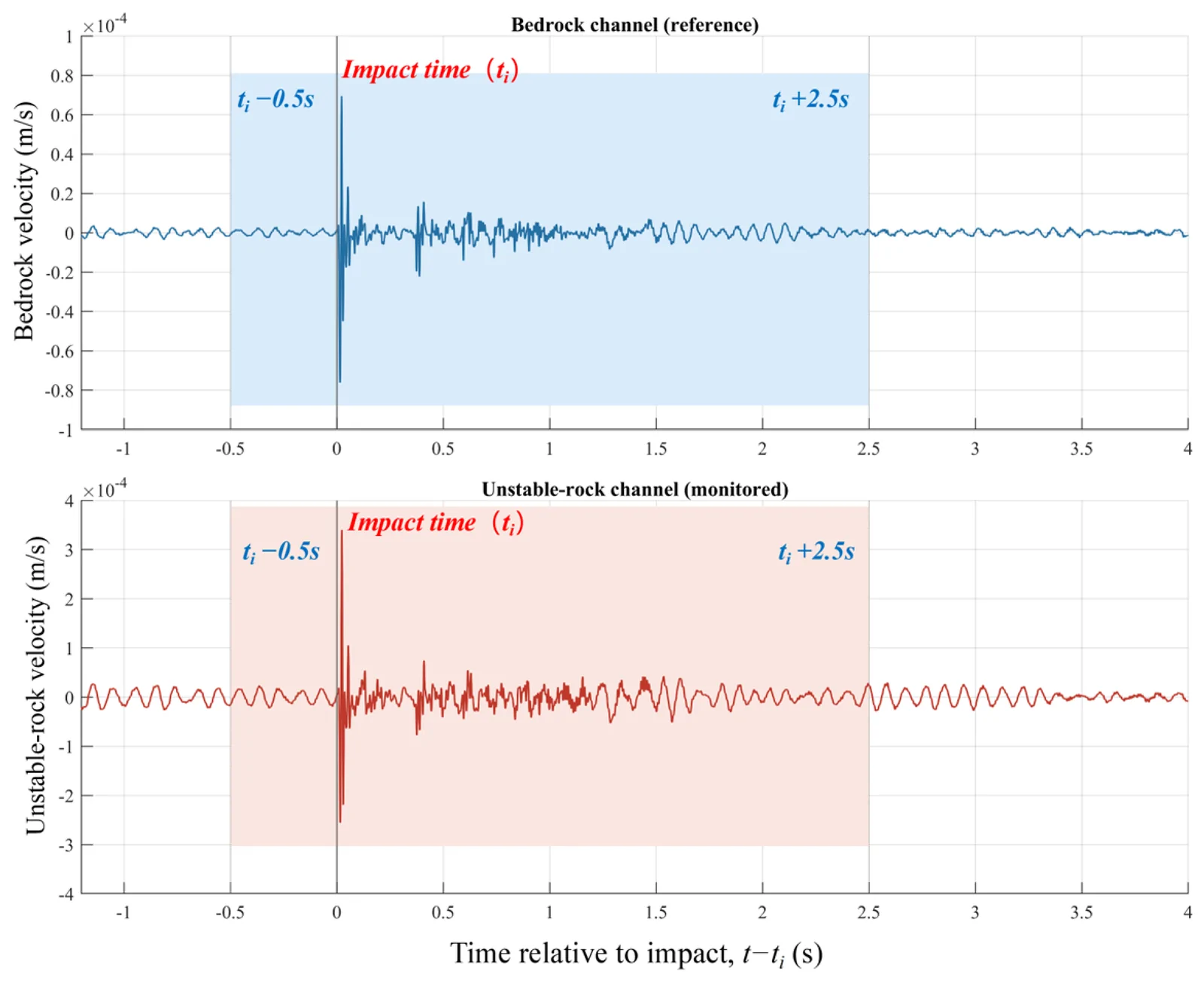
Typical impact event and schematic definition of the direct external-disturbance interval.

**Figure 5:**
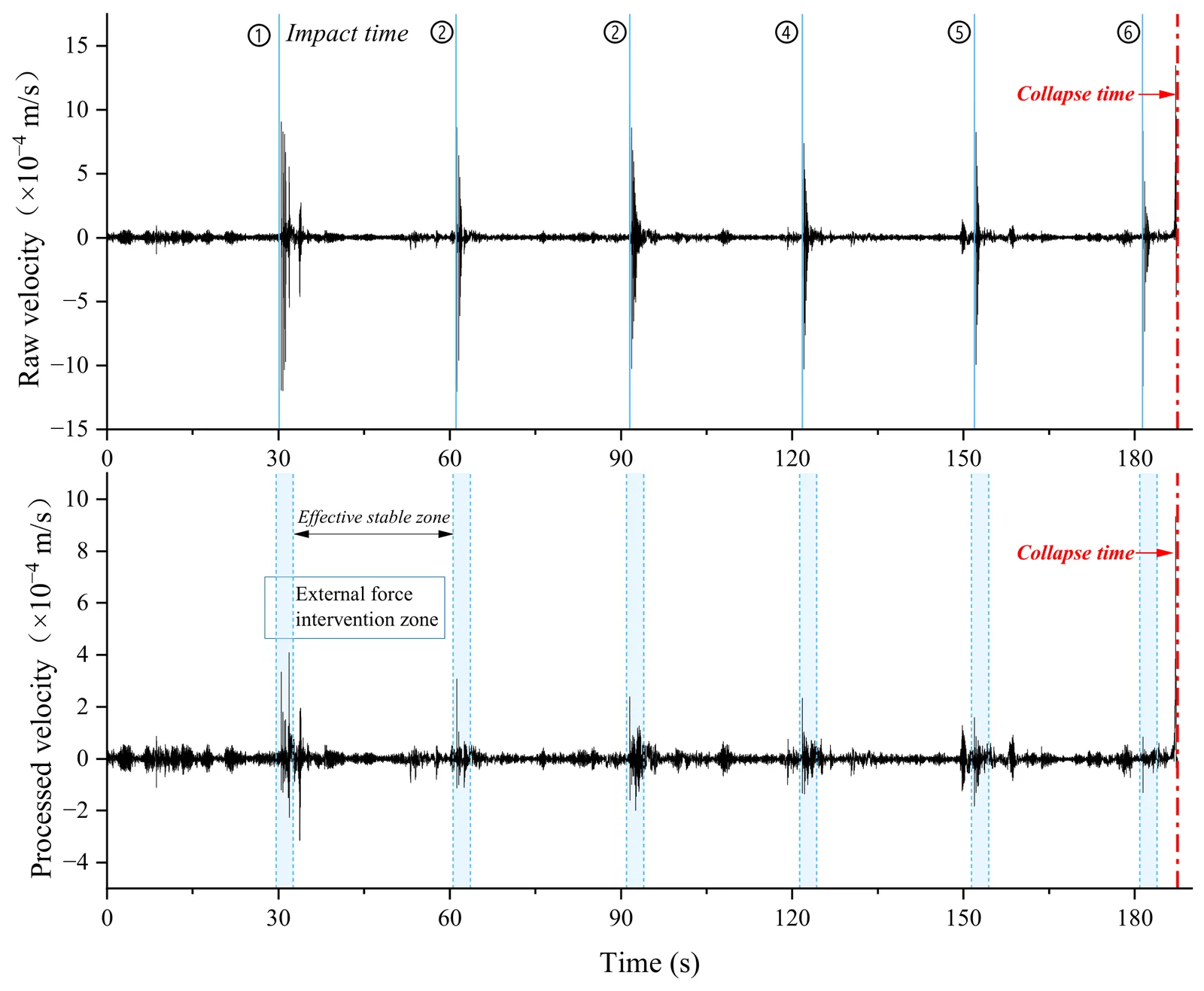
Typical raw and preprocessed vibration velocity time histories of the bedrock and unstable rock, with identification of the direct external-disturbance interval and effective analysis interval.

It can be observed from the raw time-domain signals that each ball impact produced an obvious transient pulse response in both monitoring channels of the bedrock and the unstable rock mass (Figure 4), and the pulse peaks all appeared near the manually marked ball-impact moments, indicating that the external impact acted on both the bedrock and the unstable rock simultaneously and formed vibration responses with a clear temporal correspondence at the two monitoring positions. Since the ball impact is a directly applied external force, the transient high-amplitude signal it produced mainly reflects the external excitation input and its rapid propagation process, rather than the free response of the unstable rock in the non-loading state. According to the duration range of the impact transient response in the raw time-domain signals, this study defined the interval from 0.5 s before each ball-drop impact to 2.5 s after the impact as the direct external-disturbance interval (Figure 5) and excluded it in subsequent feature calculations.

As the number of aftershocks increased, the unstable rock channel in some tests also gradually showed increasing amplitude during the non-loading stages, reflecting that, in addition to the ball-impact transient response, the background vibration level of the unstable rock itself also changed. In contrast, the bedrock channel remained relatively stable over most of the effective analysis intervals and could provide a relatively continuous background reference for the unstable rock response. Therefore, subsequent analyses were all carried out around the effective analysis segments outside the direct external-disturbance intervals, so as to identify the long-term response evolution of the unstable rock under the dynamic disturbance of multiple simulated aftershocks.

#### 3.1.2. Analysis of *RMS* and Peak Evolution

The root mean square (*RMS*) is an important time-domain parameter describing the overall intensity of a vibration signal. It can comprehensively account for the amplitude variation in the signal over a certain time interval and is more suitable than a single instantaneous peak for characterizing the average vibration level of an entire effective analysis segment. For a discrete vibration signal *x_i_*, the *RMS* is defined as

(5) $$RMS=\sqrt{\frac{1}{N}\sum_{i=1}^{N}x_{i}^{2}}$$

where *x_i_* is the vibration amplitude at the *i-th* sampling point, and *N* is the number of sampling points within the effective analysis interval.

In addition to the *RMS*, which describes the average vibration energy over an entire segment, the peak absolute vibration velocity (*PAV*) is introduced to characterize the instantaneous shock intensity. For a discrete vibration signal *x_i_*, the *PAV* is defined as the maximum absolute amplitude within an effective analysis segment:

(6) $$PAV=\max\left|x_{i}\right|,i=1,2,\cdots ,N$$

To reduce the influence of differences in the initial vibration level among test groups on result comparison, normalized *RMS* was further used to describe the degree of change in each stage relative to the initial stable state. In this study, the arithmetic mean of the *RMS* of the first three effective quiet segments at the beginning of each test was taken as the stable baseline, i.e.,

(7) $$RMS_{\mathrm{baseline}}=\frac{1}{M}\sum_{j\in \mathrm{baseline}}RMS_{j}(M=3)$$

The normalized *RMS* of the *i-th* effective analysis segment was then defined as

(8) $$RMS_{i}^{※}=\frac{RMS_{i}}{RMS_{\mathrm{baseline}}}$$

Analogous to the normalized *RMS*, a normalized *PAV* was used to remove the influence of different initial shock levels among test groups. Taking the arithmetic mean of the *PAV* of the first three effective quiet segments of each test as the stable baseline, the normalized *PAV* of the *i-th* segment is

(9) $$PAV_{i}^{※}=\frac{PAV_{i}}{PAV_{\mathrm{baseline}}}$$

The same first-three-segment baseline and normalization are used for both *RMS* and *PAV*, so that their normalized values are directly comparable within each group.

**Figure 6:**
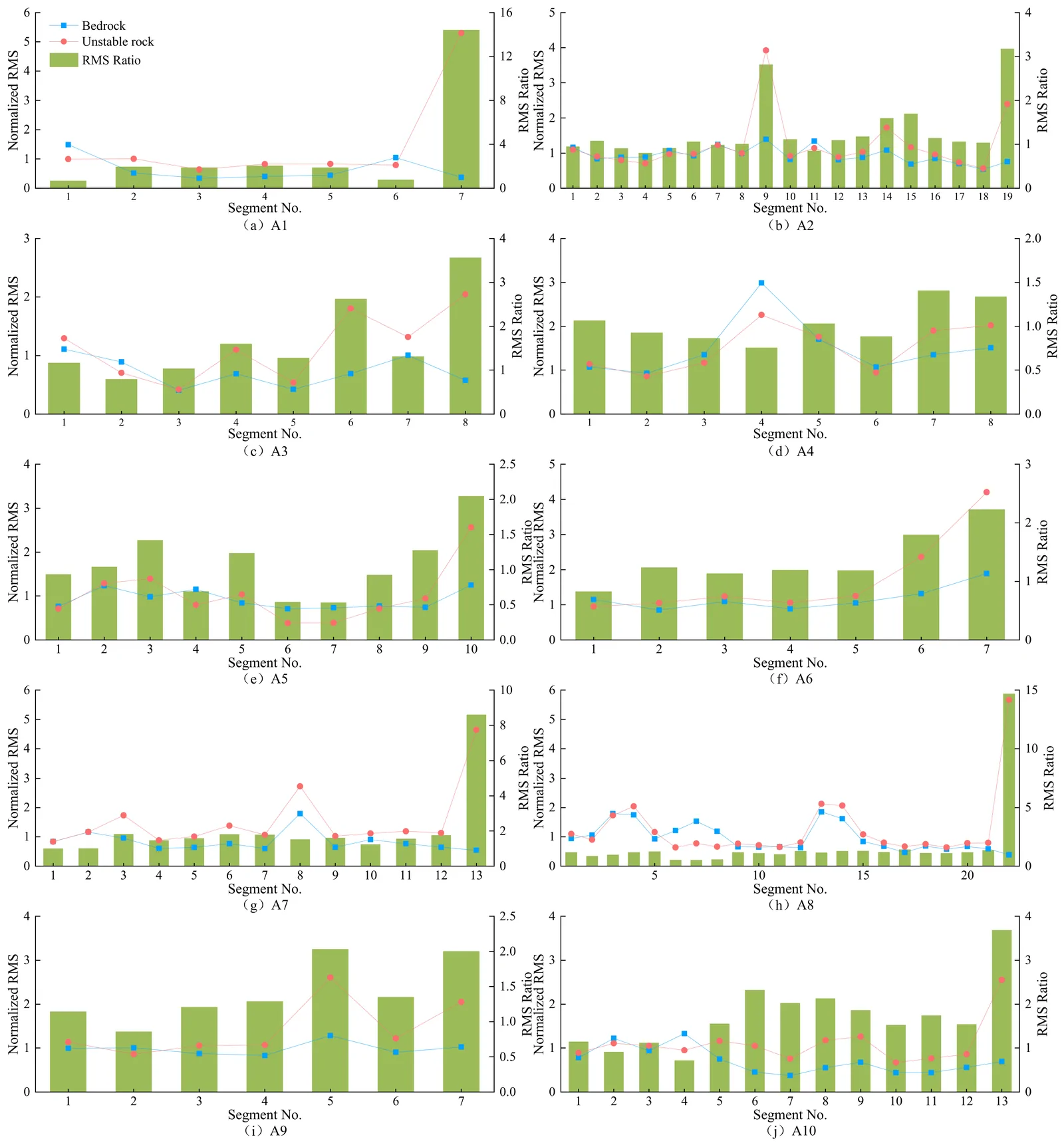
Evolution of normalized *RMS* and *RMS* Ratio of the bedrock and unstable rock.

Figure 6 shows the variation in the normalized *RMS* and *RMS* Ratio of the bedrock and the unstable rock mass with the sequence number of the effective analysis segments for groups A1–A10. Overall, the normalized *RMS* of the unstable rock mass in all ten test groups exhibited staged fluctuations of varying degrees, with the overall evolutionary direction showing an increasing trend, and most gradually developed toward a higher vibration level on the basis of local declines and recoveries. It can be seen that the enhancement of the unstable rock response under multiple simulated aftershock impacts is clearly intermittent and staged rather than a simple continuous linear increase. Some test groups showed local enhancement in the early-middle stage and then fell back to a lower level, whereas other groups showed a more concentrated rapid increase in the later stage, finally forming a vibration response significantly higher than that in the earlier stage. In the later stage of the test, the normalized *RMS* of the unstable rock mass in almost all groups rose substantially.

Looking at the specific curves of each group, the later-stage enhancement of A1 was the most prominent: its normalized *RMS* was at a relatively low level in the first few analysis segments and then showed an obvious jump in the stage approaching collapse; A2 exhibited strong inter-segment fluctuations, with a relatively obvious local enhancement in the middle stage followed by a decline and another increase near collapse; A3 changed relatively gently overall, and although its vibration level increased in the later stage, it did not show a jump of the same degree as the high-amplitude groups; A4 and A5 were generally at an intermediate level, with curves showing later-stage enhancement after multiple fluctuations; A6 changed little in the early stage and gradually rose in the second half; both A7 and A8 showed relatively obvious staged growth, with responses near collapse significantly higher than in the earlier stage; A9 had a relatively low overall amplitude level but still showed a certain degree of enhancement in the later stage; and A10 showed a relatively obvious later-stage upward trend. Overall, there were clear inter-group differences in the *RMS* evolution among test groups: some tests had small fluctuations in the early stage and rose rapidly in the later stage, whereas others showed anomalous enhancement in the middle stage followed by a brief decline and another increase before collapse. Although the specific evolutionary paths were not entirely consistent, most tests showed the general trend of the unstable rock vibration response developing from an initial stable state toward later-stage enhancement, and the enhancement was more obvious in the stage approaching collapse.

**Figure 7:**
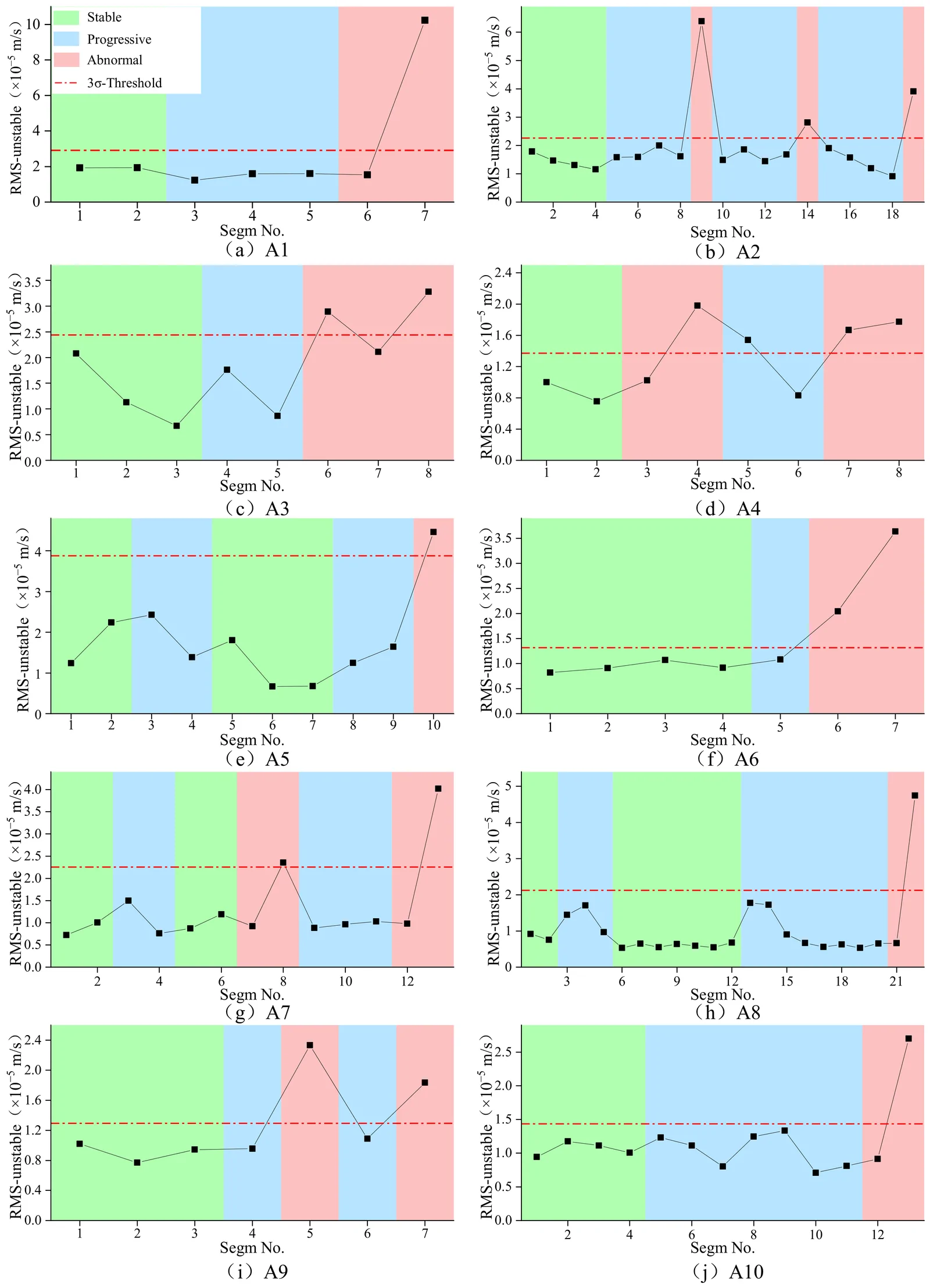
Evolution of unstable rock *RMS* and stage classification based on the 3σ reference upper limit.

Figure 7 presents the evolution of the unstable rock *RMS* with the sequence number of effective analysis segments and the stage classification results for A1–A10. The red dashed line indicates the 3*σ* reference upper limit calculated from the three stable effective analysis segments at the beginning of the test, which can characterize the degree to which the *RMS* deviates from the initial stable state; the first exceedance of this reference upper limit is treated only as a candidate anomaly signal. The three background regions in the figure correspond to the stable stage (*RMS* ≤ *μ* + *σ*), the progressive degradation stage (*μ* + *σ* < *RMS* ≤ *μ* + 3*σ*), and the imminent-collapse stage (*RMS* > *μ* + 3*σ*), respectively. From the overall evolution, the unstable rock *RMS* in each group fluctuated slightly around the baseline at the beginning of the test without significant deviation, corresponding to the stage of intact interface bonding and stable structural response; as the number of aftershocks increased, the *RMS* in some groups rose intermittently and exceeded the 3*σ* reference upper limit, which can be regarded as an anomaly signal of the vibration response deviating from the initial stable state, and if this anomaly persisted in subsequent effective analysis segments and was consistent with changes in other dynamic indicators, it could serve as an auxiliary criterion for the interface degradation stage; near the final collapse, the unstable rock *RMS* in each group showed a persistent, substantial jump, continuously breaking through the 3*σ* threshold and reaching several times the earlier baseline, presenting typical dynamic response characteristics of imminent collapse. Inter-group comparison shows that the specific evolutionary paths of different tests had certain heterogeneity: A1, A5, A6, A7, A8, and A10 showed an evolutionary pattern of rapid steep increase in the later stage, while the other three groups presented a pattern of multiple rounds of fluctuating increase, but all tests followed the common evolutionary law of a stable baseline, intermittent anomalies, and significant amplification near collapse.

#### 3.1.3. Comparison Between *RMS* and *PAV*

Across the 114 effective segments, the normalized *PAV* and normalized *RMS* were strongly but not perfectly correlated, confirming that the two indicators describe the same overall intensity trend with different sensitivity. The peak normalized *PAV* over the life of each specimen ranged from 4.52 to 18.52, markedly wider than the peak normalized *RMS* range of 2.14 to 6.03, and in nine of the ten groups, the *PAV* peak exceeded the *RMS* peak. This difference is consistent with the physical meaning of the two quantities: the *PAV* responds to isolated sharp impulses generated as the interface approaches rupture, whereas the *RMS* is averaged over the segment and therefore rises more gradually.

**Figure 8:**
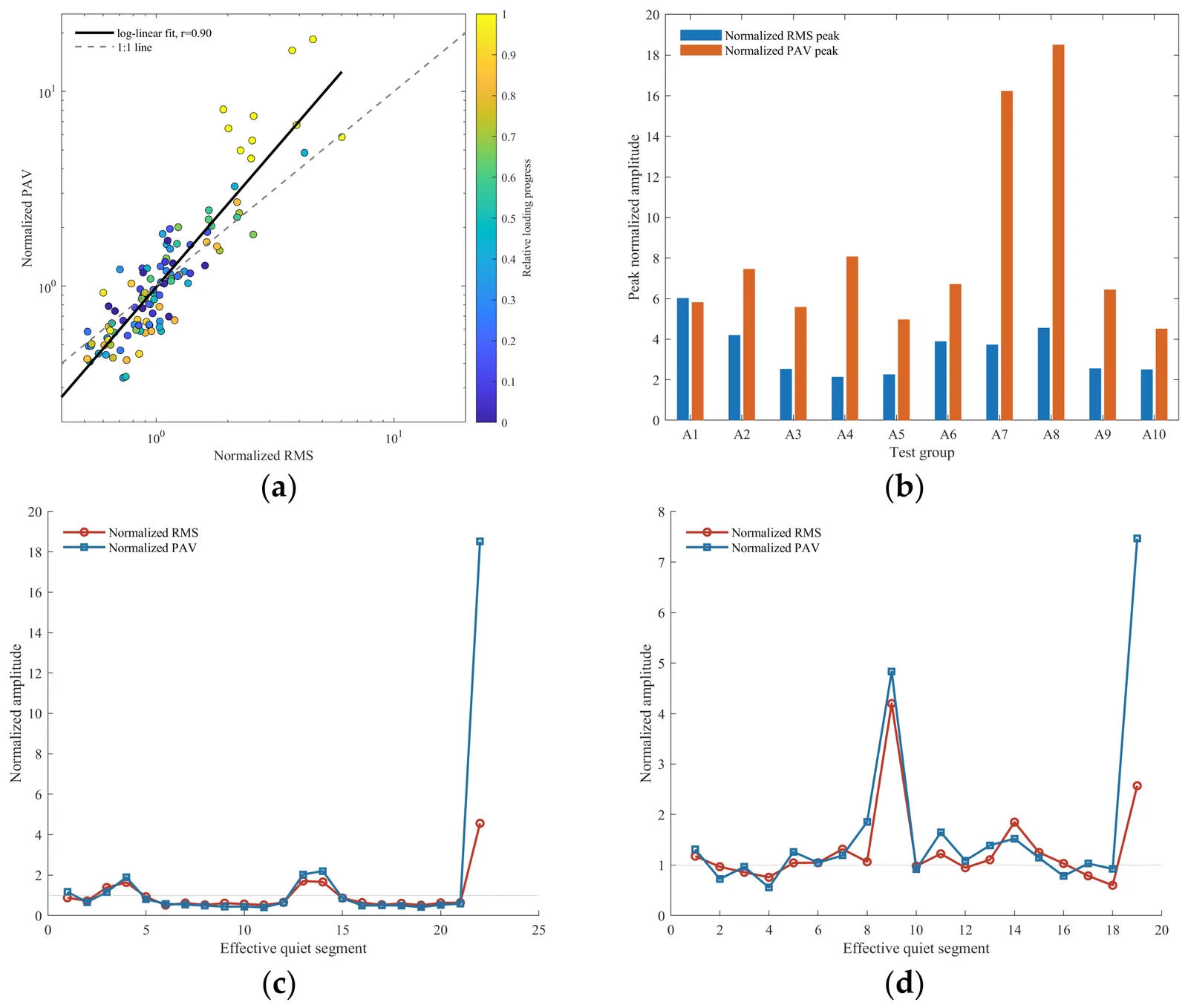
*PAV* vs. *RMS*. (**a**) Segment-level correlation on log–log axes (color: relative loading progress); (**b**) peak normalized *RMS* and *PAV* of the ten specimens; (**c**) A8 concurrent end-of-life rise (the gray dashed line denotes the 1:1 reference line); (**d**) A2, where the *PAV* isolates the collapse segment while the *RMS* shows a mid-test false peak (the gray dashed line denotes the 1:1 reference line).

Most importantly, the *PAV* peak of all ten specimens occurred at the very last effective segment, i.e., immediately before collapse, whereas the *RMS* peak was displaced to an earlier middle segment in three specimens (A2, A4, A9). In those three groups, the single-*RMS* criterion therefore produced an early “false peak” that could be misread as an imminent-collapse signal, while the *PAV* continued to increase monotonically and identified the collapse segment correctly. The *PAV* was also statistically more variable: its segment-wise coefficient of variation exceeded that of the *RMS* in most groups (e.g., A8: 2.33 vs. 0.89; A7: 2.02 vs. 0.69), and the mean crest factor increased from about 5.5 in the stable and progressive stages to 8.5 in the pre-failure stage (with a much larger standard deviation of 6.1), indicating that the pre-failure waveform became increasingly dominated by a small number of sharp impulses. These results show that the *RMS* and *PAV* are complementary: the *RMS* provides a stable measure of the overall energy level and is well suited to defining a statistical baseline, whereas the *PAV* is more sensitive to the transient, impulsive character of the imminent-collapse response and helps suppress non-terminal “false peaks” that arise from unusually large single impacts (Figure 8).

### 3.2. Evolution of Frequency-Domain Characteristics

Time-domain indicators can reflect changes in vibration intensity. To further analyze the changes in the dynamic properties of the bedrock and the unstable rock mass under multiple simulated aftershock impacts, this study performed a fast Fourier transform (*FFT*) on the preprocessed signals within each effective analysis segment and extracted the peak frequency and its corresponding spectral peak amplitude.

(10) $$X(k)=\sum_{n=0}^{N-1}x(n)e^{-j2\pi k\frac{n}{N}}$$

where *x*(*n*) is the discrete time-domain vibration signal, *N* is the total number of sampling points, and *X*(*k*) is the corresponding frequency-domain signal.

According to the spectral amplitude, the frequency with the maximum amplitude within the specified analysis frequency band was determined as the peak frequency *f_p_*, and the spectral amplitude at this frequency was defined as the peak amplitude *A_p_*. Therefore, the peak frequency mainly represents the dominant frequency characteristic of the current vibration response, while the peak amplitude reflects the relative response intensity of this dominant frequency component.

In Figure 8, the dominant frequency within each stable segment of the unstable rock in the ten test groups was overall concentrated near 10–11 Hz, with group means of 10.58–11.17 Hz, and the dominant vibration frequencies of different groups were generally close. Further statistics over all effective analysis segments showed that about 94% of the dominant frequencies of the unstable rock fell within the range of 10.0–11.4 Hz; only a few segments showed local high values above 13 Hz, manifesting as short-term frequency jumps rather than forming persistent frequency migration. In contrast, the peak frequency of the bedrock was generally higher than that of the unstable rock, with group means of approximately 11.78–13.15 Hz. In the stage approaching collapse, the directions of change in the dominant frequency were not consistent among groups; therefore, under the conditions of this test, the dominant frequency did not exhibit a unified monotonic evolution law, and its indicative role for the imminent-collapse state was relatively limited.

**Figure 9:**
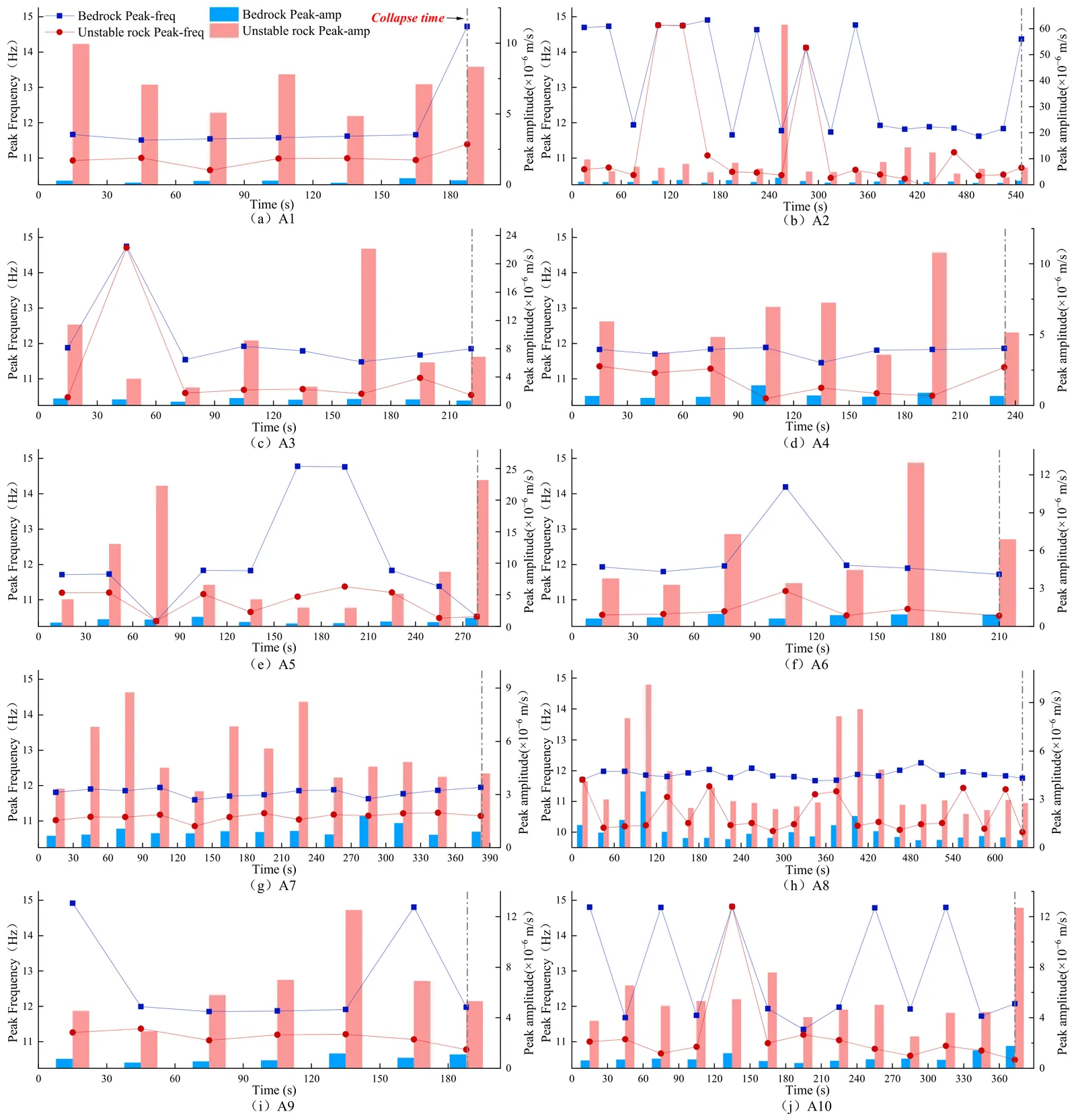
Evolution of peak frequency and spectral peak amplitude of the bedrock and unstable rock.

This study extracted the peak amplitude corresponding to the dominant frequency from the spectrum of each effective analysis segment and compared its changes during multiple simulated aftershock impacts. As shown by the bar charts in Figure 9, the peak amplitude of the unstable rock mass was overall significantly higher than that of the bedrock and exhibited relatively obvious inter-group and inter-segment differences. The peak amplitudes of the unstable rock in each group did not show strictly monotonic growth but rather fluctuated, with relatively obvious enhancement in the later stage of some tests. Compared with the dominant frequency, changes in peak amplitude more directly reflected changes in the vibration response intensity of the unstable rock. Especially in the stage approaching collapse, the spectral peak amplitude of the unstable rock in most groups showed varying degrees of elevation, in good agreement with the *RMS* enhancement phenomenon in Section 3.1.

There were certain differences in the absolute peak amplitude among test groups, so simply comparing absolute amplitudes could not fully reflect the degree of structural evolution of each group. However, from the within-group changes, the enhancement of peak amplitude in the later stage was relatively common, indicating that, with the accumulation of multiple simulated aftershock impacts, the response intensity of the dominant vibration component of the unstable rock tended to increase overall.

### 3.3. Dual-Channel Coupling Response

To further distinguish the external dynamic input from the local structural response of the unstable rock, this study, on the basis of synchronous monitoring of the unstable rock and the bedrock, introduced dual-channel coupling indicators to characterize the dynamic relationship between the bedrock and the unstable rock from three aspects: response intensity difference, vibration synchronism, and time delay and waveform distortion. Multi-indicator joint analysis can avoid judging the dual-channel dynamic relationship by relying solely on a single amplitude indicator.

#### 3.3.1. Amplitude Divergence Characteristics of the *RMS* Ratio

To reduce the influence of external ambient vibration on the identification of anomalous unstable rock responses, the bedrock vibration response was used as the ambient background reference, and the ratio of the unstable rock *RMS* to the bedrock *RMS* was defined as the *RMS* Ratio. This indicator is used to characterize the relative vibration amplification of the unstable rock mass relative to the bedrock background, and its calculation formula is shown in the following equation:

(11) $$R_{RMS}=\frac{RMS_{\mathrm{unstable}}}{RMS_{\mathrm{bedrock}}}$$

where *RMS*_unstable_ and *RMS*_bedrock_ are the *RMS* values of the unstable rock and the bedrock within the same effective analysis segment, respectively.

The green bar curve in Figure 6 shows that the *RMS* Ratio overall tended to increase as the test progressed, but its inter-group dispersion was significantly higher than that of the normalized *RMS*, and the evolutionary amplitudes of groups such as A1 and A2 differed greatly, indicating that the dynamic amplification of the unstable rock relative to the bedrock did not remain at a fixed level but changed in stages during the test. Specifically, A1 showed the most significant *RMS* Ratio growth at the end; although A2 fluctuated greatly throughout, its *RMS* Ratio in the final stage was still higher than in the earlier stage; the absolute *RMS* enhancement of A3 was not prominent, but its *RMS* Ratio still showed an obvious elevation in the later stage; A4, A5, and A6 showed staged fluctuations and later-stage enhancement of varying degrees; A7 and A8 likewise showed a large *RMS* Ratio in the later stage; and A9 and A10 had relatively low overall vibration amplitudes, but their *RMS* Ratios still maintained an increasing trend.

It should be noted that the *RMS* Ratio and the normalized *RMS* of the unstable rock did not always change synchronously. In some groups, a high unstable rock *RMS* already appeared in the middle stage, but the corresponding *RMS* Ratio did not reach its maximum; on the other hand, in some groups, the absolute *RMS* increase was limited, but because the bedrock background response was low, the *RMS* Ratio could still show obvious growth. This result indicates that the normalized *RMS* and the *RMS* Ratio reflect, respectively, the vibration intensity of the unstable rock itself and its response difference relative to the bedrock background; the two are clearly complementary, and a single indicator can hardly fully describe the dual-channel response evolution.

In the stage approaching collapse, most groups showed synchronous elevation of the normalized *RMS* and the *RMS* Ratio; that is, in the final failure stage, the unstable rock not only had enhanced vibration intensity itself, but the amplitude difference with the bedrock background vibration also further widened, which makes the *RMS* Ratio an important relative indicator for characterizing the enhancement of the local response of the unstable rock.

#### 3.3.2. Statistical Characteristics of the Sliding Pearson Correlation Coefficient

The *RMS* ratio only reflects the relative difference in vibration amplitude between the two monitoring channels, but cannot characterize the synchrony of their waveform variations. Accordingly, the sliding Pearson correlation coefficient was adopted in this study to quantify the linear correlation between bedrock and unstable rock vibration signals within the same time window (Equation (12)). Compared with analyzing the vibration amplitude of unstable rock alone, this indicator captures the consistency of vibration responses between the two channels over time.

After sliding-window calculation, the full-test time series of correlation coefficients was obtained. The median value of correlation coefficients within each effective analysis segment was selected as the primary statistic (less sensitive to local anomalies and extreme fluctuations), with the mean value as an auxiliary reference:

(12) $$r=\frac{\sum_{i=1}^{N}\left(x_{i}-\bar{x})(y_{i}-\bar{y}\right)}{\sqrt{\sum_{i=1}^{N}{\left(x_{i}-\bar{x}\right)}^{2}}\sqrt{\sum_{i=1}^{N}{\left(y_{i}-\bar{y}\right)}^{2}}}$$

where *x_i_* and *y_i_* are the vibration signal sequences of the bedrock and the unstable rock, respectively, and $\bar{x}$, $\bar{y}$ are the means of the corresponding sequences.

**Figure 10:**
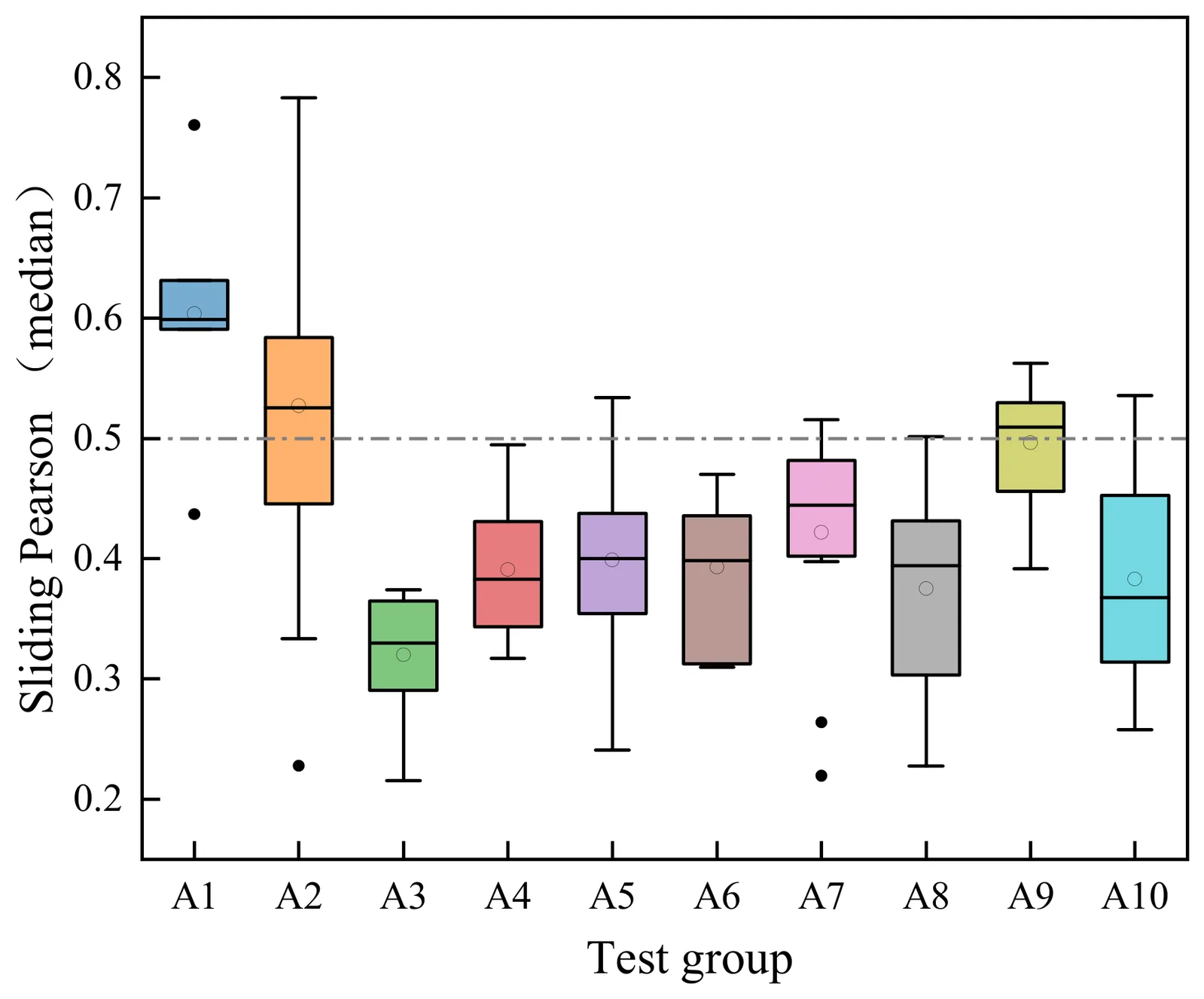
Boxplot of the distribution of sliding Pearson correlation coefficients.

As shown in Figure 10, notable inter-group differences exist. The median values across the ten groups ranged from 0.320 to 0.604, with Group A1 the highest and Group A3 the lowest. The difference between the mean and median was generally below 0.03 in each group, indicating stable internal distributions without severe skewness. Groups A1, A2 and A9 maintained relatively high correlation levels, while Group A3 showed overall weak synchrony. For intra-group distribution, Group A1 was highly concentrated around its median, whereas Group A2 had a wide distribution range, reflecting obvious stage-wise fluctuations in dynamic synchrony.

The correlation coefficient presented distinct stage-dependent changes with no uniform monotonic trend. Several groups (e.g., A2, A6, A7) underwent a decline in correlation at the late test stage, indicating weakened synchronous response between the two channels under successive simulated aftershocks. In contrast, some groups (e.g., A1, A5, A8, A10) showed increased correlation in the later stage. Note that a decrease in correlation only reflects reduced consistency of waveform variations and cannot independently verify a specific damage mechanism, which requires combined interpretation with other indicators.

The Pearson correlation coefficient complements the *RMS* ratio well: the former describes waveform synchrony while the latter reflects relative amplitude. Combining the two avoids confounding response amplitude amplification with dynamic synchrony variation. Overall, the sliding Pearson correlation coefficient reveals a shared dynamic response between bedrock and unstable rock, with significant inter-group differences and stage-wise variations, making it suitable for identifying the evolution of dual-channel dynamic relationships.

#### 3.3.3. Rock Cross-Correlation Analysis

**Figure 11:**
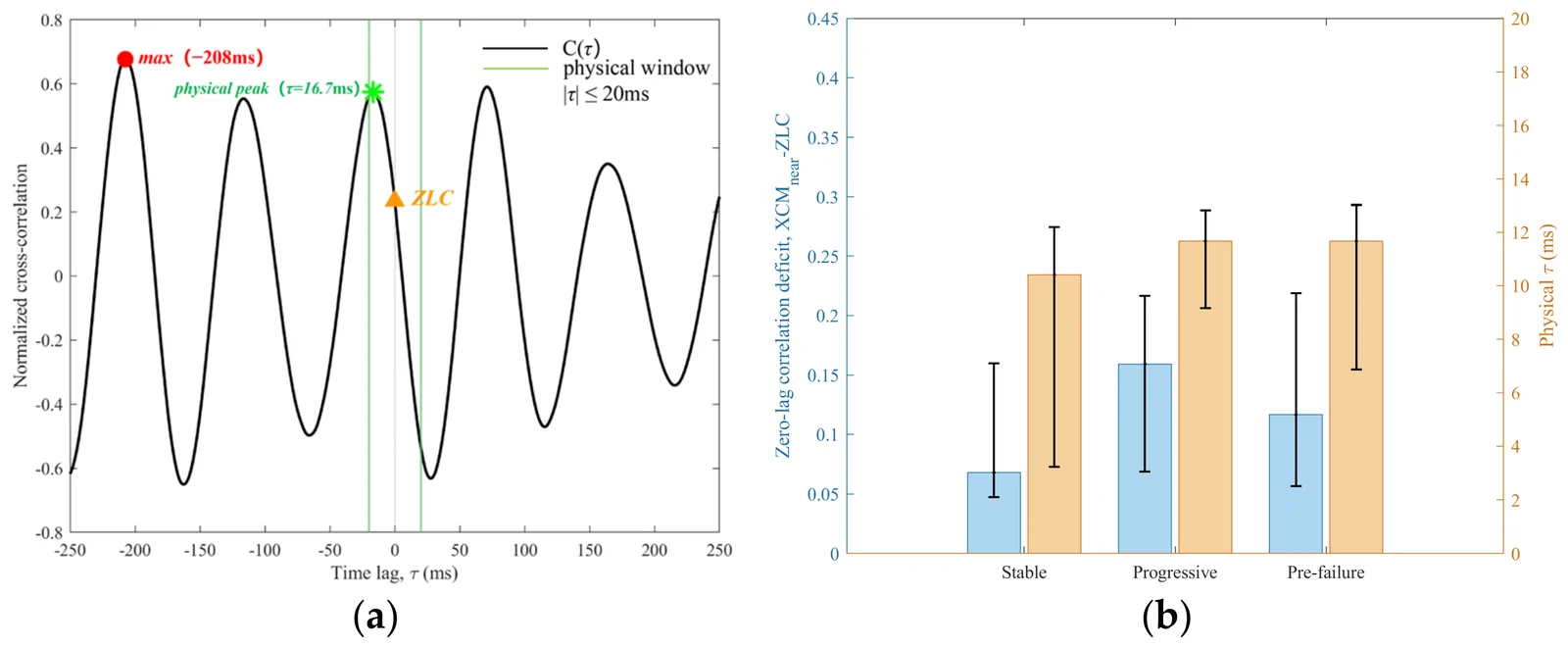
Physical time delay and coupling regimes. (**a**) Cycle skipping of the global cross-correlation (B3 segment 6): global peak (red) outside the physical window, physical near-zero peak (green) and zero-lag value (orange); (**b**) Stage-wise medians (IQR) of the zero-lag deficit and *τ_p_*.

Although the sliding Pearson correlation coefficient can characterize the linear synchrony between the two channels within a certain time window, there exists a propagation time delay for vibration transmitted from the bedrock to the unstable rock. Evaluations based only on zero-time correspondence may underestimate the actual waveform similarity between the two channels. To distinguish the effects of phase shift and waveform morphological change on correlation, this section introduces the zero-lag correlation (*ZLC*) and the near-zero-lag maximum cross-correlation (*XCM*_near_), and further extracts the physical peak time delay *τ_ρ_* to analyze dual-channel coupling evolution from two dimensions: time alignment and waveform morphology (Figure 11). For dual-channel vibration signals *x_i_* (bedrock) and *y_i_* (unstable rock) of length N, the normalized cross-correlation function is defined as:

(13) $$C_{xy}(\tau )=\frac{\sum_{i}\left(x_{i}-\bar{x}\right)\left(y_{i+\tau }-\bar{y}\right)}{\sqrt{{\sum_{i}\left(x_{i}-\bar{x}\right)}^{2}{\sum_{i}\left(y_{i+\tau }-\bar{y}\right)}^{2}}}$$

where *τ* is the time delay between the two channels.

Based on the cross-correlation function, the zero-lag correlation (*ZLC*) is defined as the cross-correlation value at *τ* = 0, i.e., the degree of linear correlation when the two signals are strictly time-aligned:

(14) $$ZLC=C_{xy}(0)$$

Within the global lag search range, the raw maximum cross-correlation (*XCM*) is defined as the global maximum of the cross-correlation function. However, it should be noted that due to the strong narrowband characteristic of the dual-channel response in this test (dominant frequency about 10–11 Hz, period about 91–100 ms), the normalized cross-correlation function itself presents an approximately equally spaced multi-peak oscillatory pattern, with peak intervals of about one dominant period. When searching for the global maximum within a wide window of ±1 s, the global optimal lag falls outside ±20 ms in 76.3% of segments (87 out of 114), often jumping to integer multiples of the dominant period such as ±68 ms or ±202 ms. This maximum is a mathematical property of the narrowband correlator and has no physical meaning as propagation time.

To obtain a physically meaningful propagation delay, the peak search is constrained to a near-zero window smaller than half the dominant period (|*τ*| ≤ *τ_max_* = 20 ms). The near-zero-lag maximum cross-correlation *XCM*_near_ and the corresponding physical peak time delay *τ_p_* are defined as:

(15) $$XCM_{\mathrm{near}}=\underset{\left|\tau \right|\leq \tau_{\max}}{\max}C_{xy}(\tau )$$

(16) $$\tau_{p}=-\arg\underset{\left|\tau \right|\leq \tau_{\max}}{\max}C_{xy}(\tau )$$

The negative sign matches the sign convention of cross-correlation (a negative lag indicates that the unstable-rock waveform lags the bedrock), so a positive *τ_p_* means that the vibration response of the unstable rock lags behind that of the bedrock. When the search window is expanded to ±30 ms, the results of *τ_p_* and *XCM*_near_ are completely consistent for all 114 segments, indicating that this indicator is insensitive to window selection and the results are robust.

Across all 114 effective segments, *τ_p_* is consistently positive (unstable-rock response lags bedrock), ranging from 1.25 to 20 ms with a median of 11.25 ms and a mean of 9.76 ms. Group-level repeatability is good, while inter-group differences mainly arise from measurement geometry and system phase characteristics. Stage-wise medians of *τ_p_* are 10.4 ms (stable), 11.7 ms (progressive degradation) and 11.7 ms (pre-failure), showing no monotonic growth with damage accumulation. Therefore, physical time delay is an inherent system property rather than a sensitive precursor of interface degradation.

#### 3.3.4. Zero-Lag Correlation Deficit and Four Coupling Regimes

With the physical delay defined, the difference between the near-zero maximum and the zero-lag correlation (*XCM*_near_ – *ZLC*) was taken as the zero-lag correlation deficit. It quantifies how much of the zero-lag similarity is recoverable after a small, physically admissible time shift, and therefore measures phase decoherence rather than a lengthening of the propagation path. The stage-wise median deficit was 0.068 in the stable stage, rose to a maximum of 0.159 in the progressive stage and fell to 0.116 in the pre-failure stage. Over the same stages, the median *ZLC* was 0.347, 0.378 and 0.351, while *XCM*_near_ was 0.515, 0.554 and 0.537. Thus, in the middle stage, the *ZLC* decreased slightly while *XCM*_near_ remained relatively high: the two channels had become phase-decorrelated under strict alignment, but their waveform similarity could still be recovered within a few-millisecond shift—this is a phase-decoherence (zero-lag-deficit) state, not a growth of the propagation delay. Only in the final collapse segments did *ZLC* and *XCM*_near_ decrease together, indicating that the waveform morphology itself had become distorted and could no longer be realigned by any admissible time shift.

**Figure 12:**
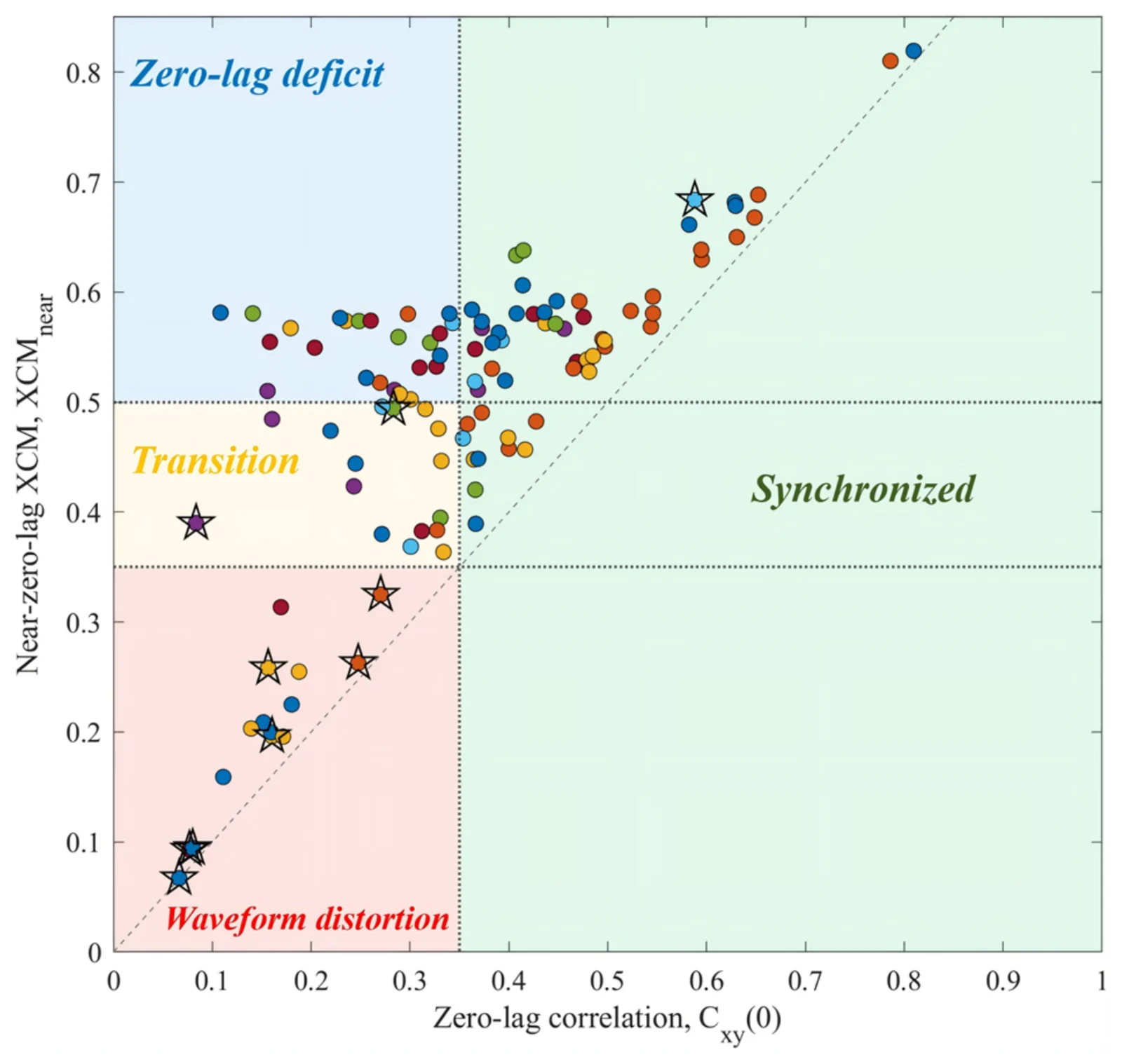
Four-regime *ZLC*–*XCM*_near_ map. The colored background zones represent four dual-channel coupling regimes: green for Synchronized, blue for Zero-lag deficit, yellow for Transition, and red for Waveform distortion.

According to the tests, using *ZLC* = 0.35 and *XCM*_near_ = 0.50 as boundaries, the dual-channel coupling state was divided into four regimes (Figure 12):

① Synchronized, *ZLC* ≥ 0.35;

② Zero-lag deficit (phase decoherence), *ZLC* < 0.35 and *XCM*_near_ ≥ 0.50;

③ Transition, *ZLC* < 0.35 and 0.35 ≤ *XCM*_near_ < 0.50;

④ Waveform distortion, *ZLC* < 0.35 and *XCM*_near_ < 0.35.

Classification of the final (collapse) segment of each specimen gave seven specimens in the waveform-distortion regime (A1, A2, A3, A7, A8, A9, A10), two in the transition regime (A4, A5, with large deficits of 0.306 and 0.211, respectively, but without recovery to the synchronized level) and one in the synchronized regime (A6, *ZLC* = 0.588, *XCM*_near_ = 0.684). The existence of a synchronized collapse specimen (A6) shows that dual-channel decorrelation, although dominant, is not a universal precursor in every specimen and must be combined with amplitude indicators rather than used alone.

## 4. Discussion

### 4.1. Controlling Effect of the Number of Simulated Aftershocks on the Degradation Process of Unstable Rock

**Figure 13:**
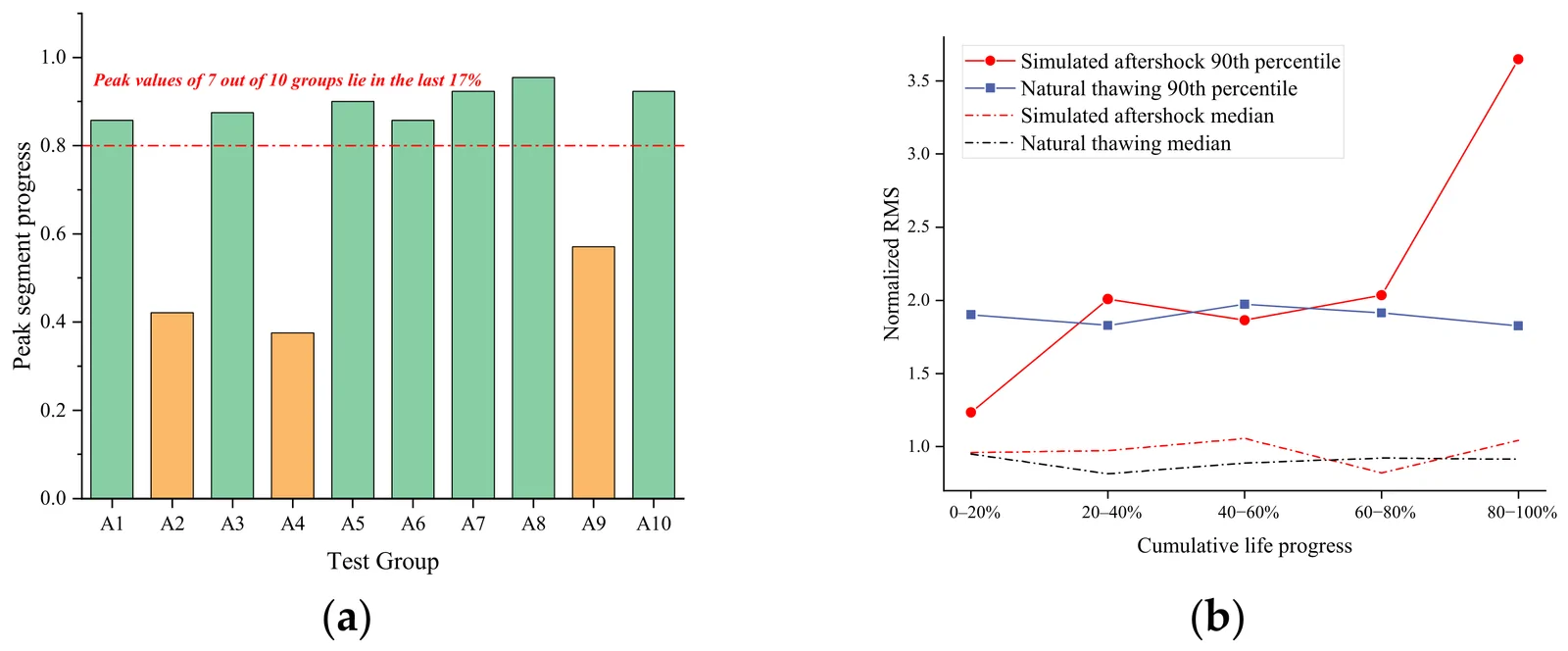
Effect of the number of ball impacts (aftershocks) on degradation: (**a**) life progress at peak response; (**b**) median and 90th percentile of normalized *RMS*.

The repeated impact tests exhibit a common failure mode of concentrated release in the terminal stage rather than smooth attenuation. To quantitatively characterize the cumulative damage effect of repeated impact loading, each effective analysis segment was matched with the cumulative number of ball drops (the *s-th* effective analysis segment corresponds to having already sustained *s-*1 times of aftershock). The analysis was carried out based on the timing of the peak response, the starting point of the 3σ anomaly, and the aftershock interval from anomaly to collapse for each group, as summarized in Table 4, combined with the temporal evolution characteristics in Figure 13.

Let *N**_eff_*** be the number of effective segments in a test and *s**_p_*** the one-based index of the segment containing the peak normalized *RMS*. Then, *n_p_* = *s_p_* − 1 and *P**_p_*** = *s_p_*/*N_eff_* = (*n_p_* + 1)/*N_eff_*. For groups in which a first 3σ anomaly was identified, the number of subsequent impacts is *N-n_3σ_*. These are counts of impacts already applied before the relevant quiet segment, not additional independent impacts occurring inside the segment.

From the distribution of the peak response, the peak normalized *RMS* of seven groups appeared concentrated in the last 20% of the entire test (peak progress 0.83–0.95), before which the normalized *RMS* remained stable for a long time at a baseline level of 0.9–1.1. This indicates that most unstable rock masses had a relatively high tolerance to repeated aftershocks, and after interface damage accumulated to the 3σ threshold, it was released in a concentrated manner as a sharp single-segment pulse of 1.91–6.45 times, presenting typical end-of-life threshold abrupt-change characteristics. A few groups (A2, A4, A9) showed peaks relatively early (peak progress 0.38–0.57); among them, A4 showed a 3σ anomaly after only one aftershock, and A2 showed an anomaly after five aftershocks, but these early anomalies did not directly trigger collapse, indicating that the early responses were dominated by recoverable phase shifts or local instantaneous disturbances and had not yet formed irreversible structural damage.

By analyzing the precursor window characteristics, a 3σ-detectable *RMS* anomaly could be observed in six groups, with an average of about six aftershocks from the appearance of the anomaly to collapse, while the other four groups (A1, A5, A7, A8) had no persistent 3σ precursor before collapse. Moreover, the normalized *RMS* of the final segments after the peak segment often fell back to near or even below the baseline (0.69–1.05), and collapse was mostly triggered by the next aftershock after the peak damage rather than occurring in the same analysis segment as the peak response, reflecting the discreteness and suddenness of the short-impending precursor.

In this study, the stage is described using a unified life progress

(17) $$Life\;progress=\frac{t}{T}$$

where *t* is the time corresponding to the selected moment, and *T* is the collapse time of the unstable rock in the two test groups. The statistics for each segment are then aggregated into five equal divisions of life progress.

The terminal concentration of damage is consistent with the picture emerging from ambient vibration monitoring of unstable slopes. Field studies have repeatedly shown that internal stiffness degradation can be detected by dynamic quantities before macroscopic displacement acceleration—records from Preonzo and La Praz captured internal damage before deformation became apparent [11,12], and ambient vibration surveys of collapse-prone rock masses have generally reported systematic changes in polarization and coherence before failure [7,8]. Our dual-channel sequences reproduced this temporal order at the laboratory scale.

### 4.2. Differences in Degradation Mechanism Between Simulated Aftershock and Natural Melting Conditions

To clarify the additional damage effect of repeated dynamic impacts relative to the natural degradation of the interface, a natural-melting control group without ball drops was set up; the model size, materials, and ice-melting conditions of the two groups were identical, differing only in whether repeated ball-drop impacts were applied. A short-duration brittle group (natural melting B1) and a long-duration recovery group (natural melting B2) were selected and paired with the simulated aftershock groups for comparison, and the key indicator statistics are shown in Table 5.

**Figure 14:**
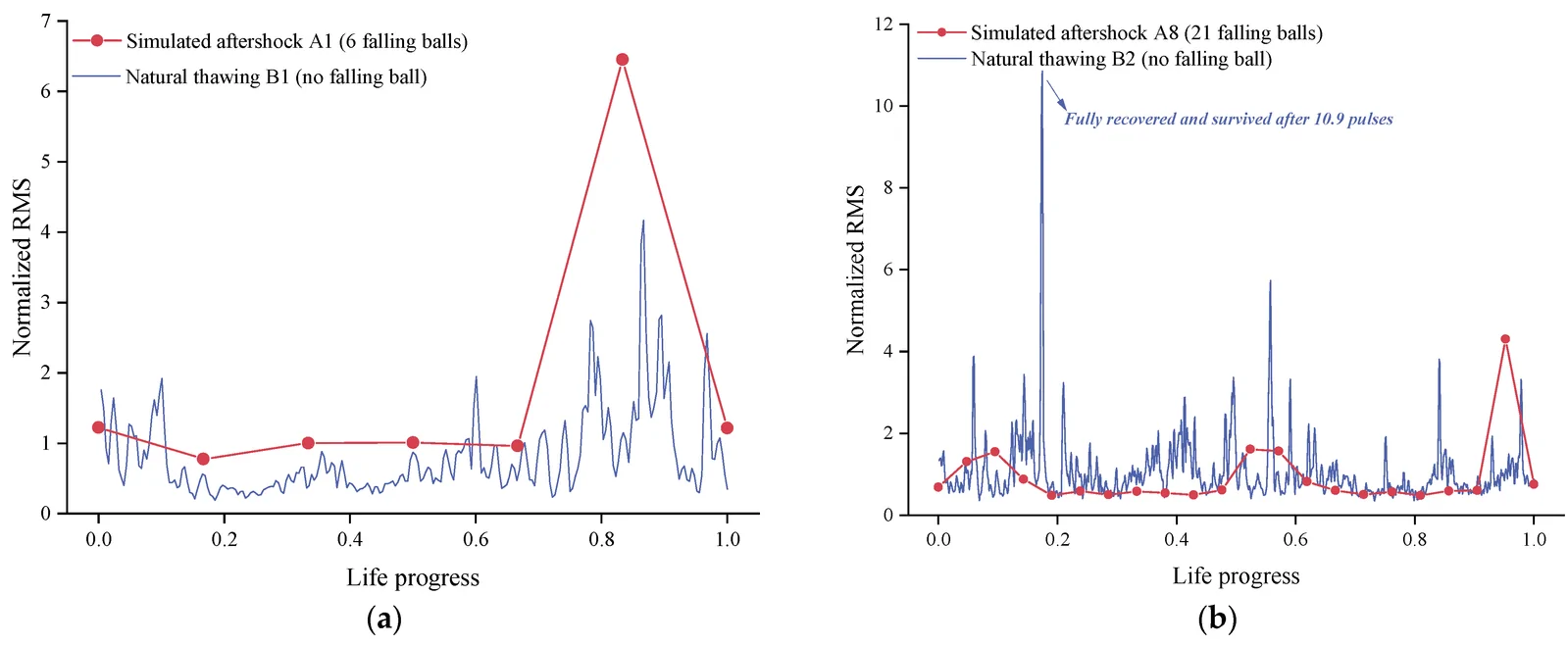
Comparison between natural melting and simulated aftershock (ball impact) conditions: (**a**) Normalized *RMS* comparison: short-duration group (about 3 min); (**b**) normalized *RMS* comparison: long-duration group (about 10 min).

From the overall statistical characteristics, the median normalized *RMS* under the two conditions was approximately constant throughout: 0.82–1.06 for the simulated aftershock group and 0.81–0.95 for the natural melting group, indicating that the average vibration levels of the two systems had no significant trend change during typical periods; however, the 90th percentile, which characterizes the strong-response level, differed significantly: the 90th percentile of the simulated aftershock group rose from about 1.2 in the early stage and about 2.0 in the middle stage to 3.65 in the final stage, while that of the natural melting group remained stable at 1.8–1.9 throughout, with no end-of-life elevation (Figure 14b). In terms of peak occurrence progress, 70% of the peaks in the simulated aftershock group were concentrated in the last approximately 17% of the life span, whereas the peaks in the natural melting group were scattered across the entire life span from 2% to 79%, with no obvious concentration trend. To statistically quantify the between-group differences in peak amplitude, peak timing, total duration and dominant frequency across all specimens, a two-sided Mann–Whitney U test was conducted between the NM (*n* = 8) and EQ (*n* = 10) groups, with descriptive statistics and test results summarized in Table 6.

The statistical analysis indicates that none of the between-group differences is statistically significant. The consistent finding is that the dominant frequency remains stable at approximately 10.8 Hz. A single large pulse should not be interpreted as evidence of irreversible damage: one specimen in the natural-melting group recovered after experiencing a pulse 10.9 times its initial value at 17% of its lifetime, whereas a specimen in the ball-drop group reached its peak at 95% of its lifetime. Therefore, persistence, rather than peak magnitude, should be the primary criterion for warning identification.

Mechanically, repeated ball drops impose discrete, high-amplitude inertial impacts that accumulate irreversible damage through interfacial opening/closing, wear, and microcracking, eventually producing threshold-type failure, hence A8’s 4.31 times peak at 95% life and A1’s 6.45 times peak after six drops. Natural melting weakens the interface continuously and slowly, allowing stress redistribution and recovery after a single disturbance; hence, B2 recovered to baseline after a 10.9 times pulse and survived about 648 s, while B1 remained stable for about 85% of life before a terminal 4.17 times pulse. The difference therefore arises from loading history rather than life progress alone: repeated impacts favor irreversible accumulation and terminal concentration, whereas continuous melting favors gradual weakening with recoverability. For monitoring, strong-response tails, peak clustering, and dynamic indicators such as propagation delay and coherence are more informative than mean or median vibration levels, though these remain laboratory-scale mechanistic findings requiring field validation.

### 4.3. Mechanism of Stationary Dominant Frequency and Applicability of Warning Indicators

Despite the continuous attenuation of interface strength and several-fold growth of vibration amplitude at the terminal stage, the dominant frequency of the unstable rock consistently concentrates within 10.0–11.4 Hz. This regularity holds for both the natural-melting and impact-loading protocols, and notably deviates from the widespread conclusion that resonant frequency declines continuously with damage accumulation in most long-term field monitoring and numerical studies.

In existing research, the mechanical premise of frequency decrease is that the monitored rock mass remains mechanically connected to the parent rock. Fracture propagation within the rock mass reduces the effective stiffness *k**_eff_***, leading to a monotonic drop in dominant frequency, following

(18) $$fn=\frac{1}{2\pi }\sqrt{\frac{k_{eff}}{m}}$$

In contrast, the test system herein features a discrete dual-block structure: the unstable rock and bedrock are two geometrically intact independent blocks weakly bonded only by a thin ice interface. Throughout the test, the block itself undergoes no fracturing, with its mass and bulk stiffness essentially constant, and degradation is confined exclusively to the interface layer. The 10–11 Hz spectral peak therefore represents the inherent vibrational property of the unstable rock block itself, governed by its mass and near-rigid support condition rather than the global mode of a continuously softening rock mass. Melting and impact breakage of the ice interface only modify force transmission efficiency and energy dissipation capacity without altering the bulk stiffness of the block; hence, the stationary dominant frequency aligns with the established inverse block-size–frequency relationship [24].

The above findings delimit the applicable boundary of frequency-type precursors. For intact rock slopes with internal continuous cracking and global stiffness degradation, declining dominant frequency serves as a sensitive damage indicator. For discrete weakly constrained blocks with intact bodies and only interface degradation, the dominant frequency is approximately conserved and insensitive to interface damage; amplitude and dual-channel coupling indicators should take the leading role, with frequency only as an auxiliary parameter. Since the ice in this bonding model largely withdraws from load-bearing at the terminal stage, its degradation path differs from scenarios such as freeze–thaw that modify the overall stiffness of the structural system [19,20]. Accordingly, stationary dominant frequency cannot be used to negate progressive interface damage, and amplitude plus reference-channel coupling metrics should be prioritized for early warning of such targets.

### 4.4. Engineering Early Warning Significance of the Dual-Channel Vibration Response

**Figure 15:**
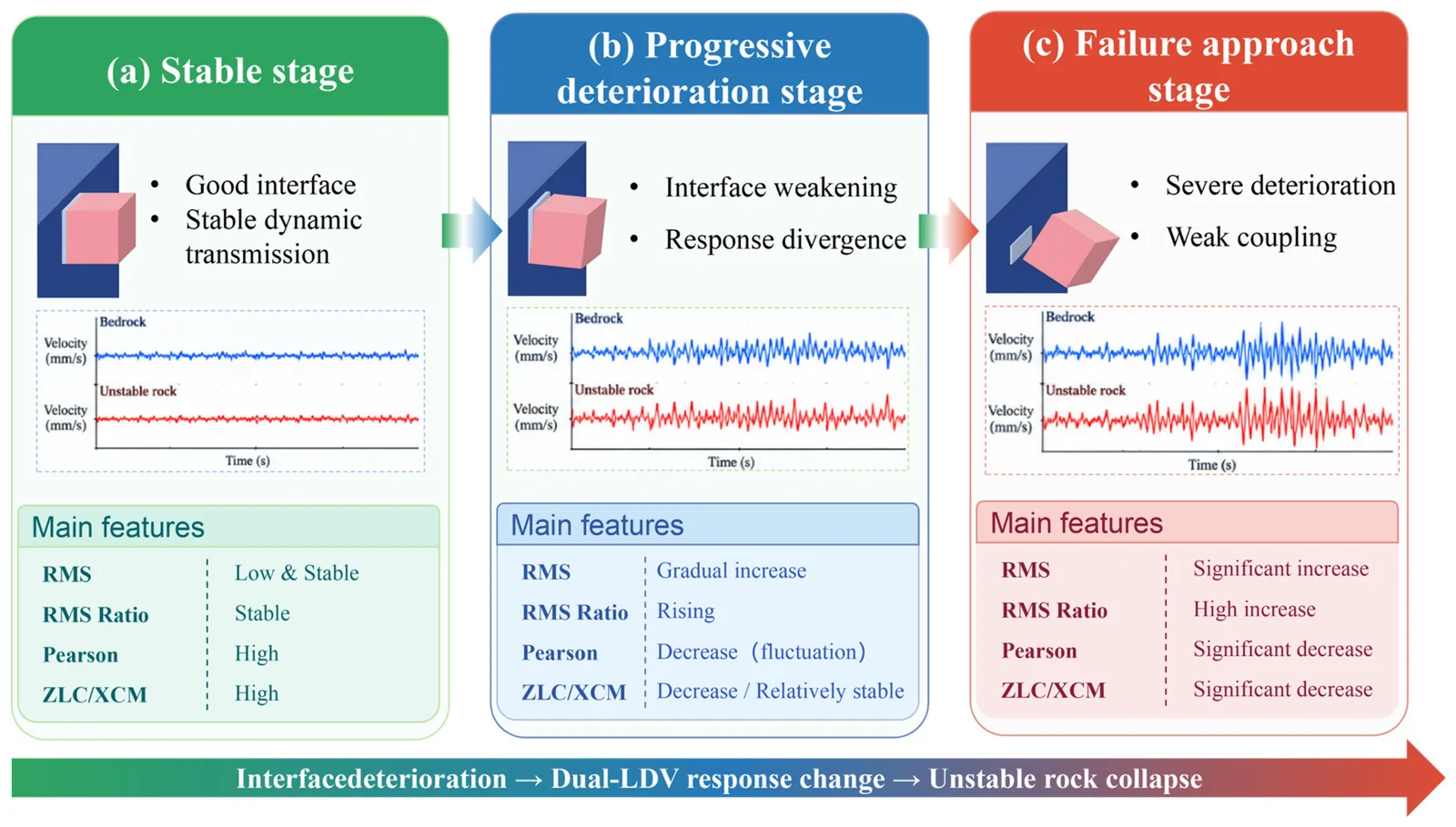
Schematic of the evolution mechanism of dual-channel vibration responses associated with interface deterioration.

**Figure 16:**
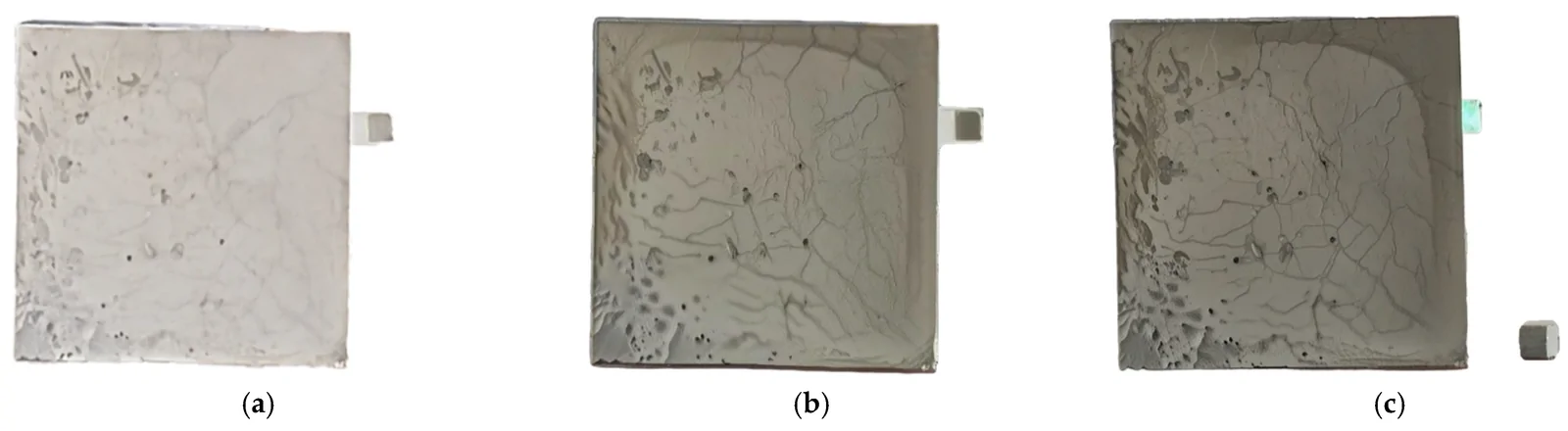
Three-stage damage evolution process of unstable rock mass under repeated impact loading. (**a**) Stable stage; (**b**) Progressive deterioration stage (sliding of unstable rock mass occurs); (**c**) Failure.

The representative physical test images of the three-stage damage evolution and the corresponding mechanism interpretation diagrams are shown in Figure 15 and Figure 16, respectively.

On the basis of the first three segments’ stable baseline of each specimen, three escalating levels were defined segment by segment:

(1) Attention (screening): N*_RMS_* > *μ* + 2*σ*, or the *RMS* ratio rises more than 30% above its baseline. This level is intentionally sensitive and is used to avoid missing an anomaly.
(2) Warning (intermediate): N*_RMS_* > *μ* + 3*σ*, and the median sliding Pearson correlation falls more than 20% below its baseline.
(3) Alarm (high-confidence confirmation): N*_RMS_* > *μ* + 3*σ*, and both *ZLC* and *XCM*_near_ fall below 0.20.

The three warning levels are evaluated segment by segment; an isolated *RMS* exceedance triggers only the Attention level, whereas the Warning and Alarm levels require their respective multi-indicator criteria to be satisfied.

The last three effective segments of each specimen were labeled as the pre-collapse window (positive class, 30 segments) and the remaining 84 segments as negative. Two single-indicator baselines were evaluated on the same labels: the single-*RMS* 3σ criterion and a single-correlation criterion (*ZLC* and *XCM*_near_ both below 0.20).

No single criterion combined high sensitivity with zero false alarms. The single-RMS 3σ criterion had a segment-level recall of 40.0% and precision of 70.6%, and it produced five false alarms in the early window of three specimens (A2, A4, A7), where an unusually large single impact transiently raised the *RMS* above 3σ but the response subsequently returned to the baseline. The single-correlation criterion was more specific (three false alarms) but missed almost all pre-collapse segments (recall 13.3%), because several specimens (e.g., A6) retained substantial dual-channel synchronism even at collapse (Table 7).

**Figure 17:**
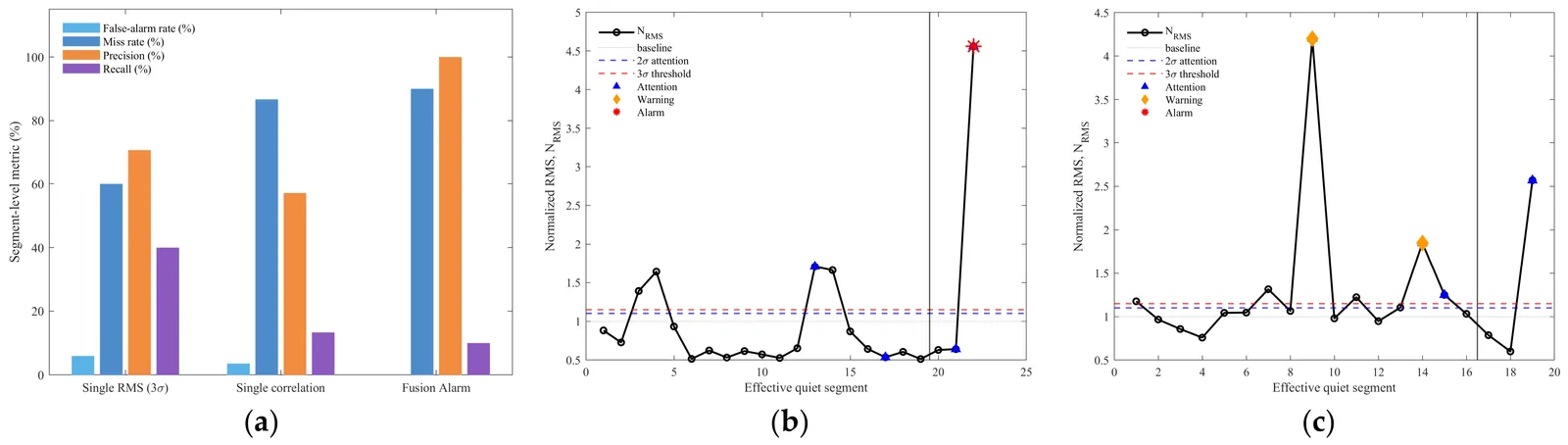
Graded fusion warning. (**a**) Segment-level performance of the five criteria; (**b**) A8 escalation to Alarm at the final segments; (**c**) A2 isolated mid-test *RMS* spike rejected by the correlation gate.

Figure 17 shows the data plots corresponding to the graded fusion warning analysis. The two fusion levels exploited complementary strengths. The stringent Alarm produced no false alarms (precision 100%): all three of its alarms were genuine pre-collapse segments, and it correctly rejected the isolated mid-test *RMS* spikes in A2, A4 and A7 because their correlation gate (*ZLC*, *XCM*_near_ < 0.20) was not crossed. In other words, fusing the 3σ amplitude condition with the dual-channel correlation condition reduced the high-severity false-alarm rate from 6.0% (five segments across three specimens) under single-*RMS* to zero. At the specimen level, Alarm identified three of the ten specimens (A1, A7, A8) with zero early false alarms, and the remaining seven specimens were not force-flagged, which is consistent with the finding that waveform distortion dominates only in a subset of failures. Conversely, the sensitive Attention level occurred in the pre-collapse window of all ten specimens (100% specimen-level window coverage), providing a screening net that compensates for Alarm’s limited segment-level recall (10%), although its higher sensitivity was accompanied by more early triggers (segment-level false-alarm rate 23.8%).

The Warning level was triggered in only four segments, indicating that a 20% drop in the sliding Pearson correlation alone is not a reliable discriminator in this small dataset. We therefore recommend a two-level operational use rather than a single fused flag: Attention as a high-sensitivity do-not-miss screening state, and Alarm as a high-specificity act-now confirmation, with the Warning level retained only as an auxiliary flag pending calibration on a larger sample.

In contrast, multi-indicator co-analysis helps distinguish short-term random fluctuations from persistent dynamic response changes and reduces the uncertainty brought by relying only on a single amplitude indicator: when only an *RMS* increase appears while coupling indicators remain stable, it is more likely an amplitude change caused by fluctuations in external excitation intensity or test noise; when the anomalous *RMS* increase is accompanied by a decrease in sliding Pearson correlation and a widening of the difference between *ZLC* and *XCM*, it corresponds to genuine development of interface damage. In addition, the traditional 3σ threshold method is based only on single-channel statistical characteristics, whereas this study combined 3σ amplitude anomalies with the co-variation in coupling characteristics, further reducing the risk of misjudgment by a single statistical criterion. ROC/AUC analysis and further validation of warning lead time using independent tests are left for future work, given the exploratory nature of the present dataset and the temporal dependence among segments from the same test.

### 4.5. Limitations and Future Work

This study still has the following limitations, which affect the applicable scope of the results.

**Model scale limitation**. The physical model is intended to examine the evolution of dual-channel vibration indicators during progressive interface degradation, rather than to reproduce a dynamically similar field rock slope. The test preserves the qualitative arrangement of a small unstable block, a much larger reference block, and a weakening interface, but does not establish similitude in prototype stress state, stiffness, damping, seismic acceleration spectrum, or impact energy. The repeated steel-ball impacts should consequently be interpreted as controlled transient disturbances, not as waveform-equivalent aftershocks. In addition, ice-layer thickness, time-dependent effective contact area, melting rate, and the mechanical parameters of the cement-mortar/ice interface were not quantified in the available experimental record. These limitations restrict direct quantitative extrapolation to natural slopes. Future work should measure specimen density and mechanical properties, control and record the interface geometry and temperature, and implement a force sensor or calibrated kinematic measurement to further examine the impact event.

**Loading method limitation.** The test used ball-drop impacts to simulate seismic loading, which is essentially a single-pulse excitation and differs from the duration characteristics and spectral composition of actual earthquakes; under dynamic loads with different spectral characteristics, the interface degradation rate and coupling response mode of the rock may differ, so relevant conclusions should be extended to real seismic loading conditions with caution.

**Degradation mechanism limitation.** The test simulated the gradual change in interface connection conditions through the natural melting of an ice block, mainly to characterize the interface degradation process under simplified conditions; this process differs from the complex damage evolution caused by weathering, water–rock interaction, and freeze–thaw cycles in natural rock, so the test results should not be directly equated with the strength attenuation law of natural structural planes.

**Qualitative nature of the natural-melting control.** The natural-melting group was not a randomized concurrent control; temperature, melting rate and initial contact area were not strictly regulated, sample sizes differ between the two protocols, and the total number of specimens is relatively limited. Accordingly, the comparison between the natural-melting (NM) and earthquake-simulation (EQ) protocols presented in Section 4.2 should be regarded as qualitative findings, which require further validation through paired, temperature-controlled experiments.

From an engineering application perspective, dual-LDV synchronous monitoring combines the technical advantages of non-contact operation, long measurement distance, and high sampling rate, and is especially suitable for engineering scenarios such as high-position unstable rock masses on high-steep rock slopes that are difficult for personnel to reach and where deploying contact sensors is difficult. Traditional displacement and crack monitoring can only obtain macroscopic deformation and has difficulty capturing the high-frequency, micro-amplitude dynamic response before collapse; in contrast, dual-LDV can deploy a bedrock reference point and an unstable rock monitoring point simultaneously from a safe distance, synchronously acquire dual-channel vibration signals, and dynamically identify the interface degradation process by extracting the co-evolution characteristics of multidimensional indicators of amplitude, frequency, and coupling. This method can serve as an important complement to traditional deformation monitoring, provide a dynamic-level monitoring basis for the early identification and graded early warning of unstable rock collapse, and has practical value for improving the dynamic monitoring capability of high-steep slopes along mountain trunk highways and water conservancy reservoir areas.

## 5. Conclusions

This study investigates the evolution of unstable-rock vibration responses under repeated impact loading using a synchronized dual-LDV monitoring system and develops a preliminary multi-indicator warning framework. The main conclusions are as follows.

(1) **Methodological contribution**. A synchronous dual-LDV monitoring scheme is developed to simultaneously measure the vibrations of the bedrock and unstable rock under non-contact conditions. By using the bedrock response as a reference, the scheme jointly characterizes amplitude, dominant frequency, and cross-channel coupling, helping distinguish shared excitation-related responses from changes specific to the unstable rock. This approach provides a practical sensing strategy for characterizing vibration evolution during progressive interface degradation.
(2) **Single-channel response pattern**. Under repeated impacts, the unstable rock exhibits a response pattern characterized by relatively stable vibration followed by late-stage amplitude amplification. In seven specimens, the peak amplitudes were concentrated within the final 20% of the observed lifespan, indicating that amplitude-related changes can provide useful information on approaching instability, although intermediate fluctuations and isolated anomalies may occur. The dominant frequency remains approximately 10–11 Hz without a consistent trend as the interface deteriorates. Under the conditions of this experiment, this stability suggests that interface degradation does not produce a substantial change in the bulk dynamic response of the unstable block, highlighting the limitations of dominant-frequency shifts as a standalone precursor.
(3) **Dual-channel coupling evolution**. The cross-channel indicators capture changes in the temporal alignment and waveform consistency between the bedrock and unstable rock during progressive interface degradation. Although the physical peak time delay is consistently positive across the analyzed segments, it does not increase monotonically with damage progression and therefore should not be used independently to quantify damage severity. The zero-lag correlation deficit primarily characterizes the loss of waveform agreement under strict temporal alignment, rather than an increase in propagation distance. These findings support the combined interpretation of correlation-based indicators while emphasizing the need to distinguish changes in waveform coupling from changes in physical propagation delay.
(4) **Damage implications and early-warning potential**. The response evolution under repeated impacts indicates the progressive accumulation of interface damage and corresponding changes in the dynamic interaction between the bedrock and unstable rock. By integrating amplitude anomalies with cross-channel coupling characteristics, the proposed graded warning framework provides a complementary approach for identifying potential precursory changes and distinguishing isolated vibration fluctuations from more coordinated response anomalies. These findings demonstrate the potential of synchronous dual-LDV monitoring for characterizing the progressive destabilization of unstable rock masses. Future work will focus on expanding the experimental conditions, refining warning thresholds, and conducting field monitoring to further improve the reliability and engineering applicability of the proposed framework.

## Figures and Tables

**Table 1:** Statistical table of natural frequencies in test specimens.

Dominant Frequency	Unstable Rock (Hz)	Bedrock (Hz)
1	11.04	12.00
2	10.91	12.02
3	11.04	11.97
4	10.62	11.90
5	11.03	12.00
Mean	10.93	11.98

**Table 2:** Dual-LDV acquisition, preprocessing and analysis settings.

Parameter	Value
LDV manufacturer	Polytec, two synchronized heads
Measurement laser	1550 nm short-wave infrared
Measured quantity	Surface vibration velocity (m/s)
Sampling frequency	2400 Hz
Velocity range	0–30 m/s (max measured peak 4.56 × 10^−4^ m/s, well below range)
Velocity resolution	8 nm/s/√Hz (nominal optimal value)
Residual inter-channel timing error	one DAQ
Spike removal	25 pt median; MAD 12-σ outliers replaced by local median
Low-pass filter	4th-order Butterworth, *f*_c_ = 150 Hz, zero-phase forward-backward filtering (filtfilt)
Detrending	Linear
Impact exclusion window	[*t_i_* − 0.5, *t_i_* + 2.5] s
Sliding Pearson window	2 s, step 1 s
Cross-correlation lag range	±1 s (global); physical *τ* within ±20 ms (identical at ±30 ms)

**Table 3:** Collapse time and total number of ball-drop impacts.

Group	Collapse Time/s	Impact Times
A1	187.5	6
A2	548.0	18
A3	221.5	7
A4	234.5	7
A5	279.5	9
A6	211.0	7
A7	383.0	12
A8	639.0	21
A9	189.0	6
A10	373.0	12

**Table 4:** Timing of the normalized-*RMS* peak and the first 3σ anomaly.

Test Group	Total Number of Ball-Drop Impacts, *N*	Number of Impacts Preceding the Peak Segment, *n_p_*	Peak Progress, *P_p_*	Number of Impacts Preceding the First 3σ-Anomaly Segment, *n_3σ_*	Additional Impacts After the First 3σ Anomaly, *N-n_3σ_*	*N_eff_*
A1	6	5	0.86	—	—	7
A2	18	7	0.42	5	13	19
A3	7	6	0.88	2	5	8
A4	7	2	0.38	1	6	8
A5	9	8	0.90	—	—	10
A6	7	5	0.86	3	4	7
A7	12	11	0.92	—	—	13
A8	21	20	0.95	—	—	22
A9	6	3	0.57	3	3	7
A10	12	11	0.92	5	7	13

**Table 5:** Comparison of key indicators between natural melting and simulated aftershock paired conditions.

Indicator	Natural Melting B1 (No Ball Impact)	Aftershock A1 (6 Ball Impacts)	Natural Melting B2 (No Ball Impact)	Aftershock A8 (21 Ball Impacts)
Collapse duration/s	248	187.5	785	639
Number of discrete ball impacts (times)	0 (melting only)	6	0 (melting only)	21
Proportion of stable stage	85%	—	12%	—
Peak normalized *RMS*/(dimensionless, × baseline (M = 3))	4.17	6.45	10.85	4.31
Peak occurrence progress (dimensionless)	0.87	0.83	0.17	0.95
Maximum *RMS* ratio (dimensionless)	182.8	204.2	21.3	55.9
Dominant frequency of unstable rock/Hz	10.90	10.99	10.14	10.58

**Table 6:** Descriptive statistics and two-sided Mann–Whitney U test for NM (*n* = 8) and EQ (*n* = 10).

Metric	EQ (Mean ± SD)	NM (Mean ± SD)	*p*	Common-Language Effect
Peak normalized *RMS*	3.44 ± 1.26	6.31 ± 3.98	0.20	0.31
Peak progress	0.85 ± 0.24	0.52 ± 0.43	0.053	0.76
Duration (s)	326 ± 158	349 ± 218	0.83	0.54
Median dominant frequency (Hz)	10.84 ± 0.30	10.84 ± 0.33	1.00	0.50

**Table 7:** Segment-level confusion matrix on 114 segments.

Method	Alarms	TP	FP	FN	TN	Accuracy	False-Alarm	Precision	Recall
Single *RMS* (3σ)	17	12	5	18	79	79.8%	6.0%	70.6%	40.0%
Single correlation	7	4	3	26	81	74.6%	3.6%	57.1%	13.3%
Fusion Warning	4	1	3	29	81	71.9%	3.6%	25.0%	3.3%
Fusion Alarm	3	3	0	27	84	76.3%	0.0%	100%	10.0%
Fusion Attention	39	19	20	11	64	72.8%	23.8%	48.7%	63.3%

## Data Availability

Some or all data, models, or code generated or used during the study are available from the corresponding author upon request.
